# Moisture-resistant, stretchable NO_x_ gas sensors based on laser-induced graphene for environmental monitoring and breath analysis

**DOI:** 10.1038/s41378-022-00414-x

**Published:** 2022-07-08

**Authors:** Li Yang, Guanghao Zheng, Yaoqian Cao, Chuizhou Meng, Yuhang Li, Huadong Ji, Xue Chen, Guangyu Niu, Jiayi Yan, Ye Xue, Huanyu Cheng

**Affiliations:** 1 State Key Laboratory of Reliability and Intelligence of Electrical Equipment, School of Health Sciences and Biomedical Engineering, Hebei University of Technology, Tianjin, 300130 China; 2 School of Mechanical Engineering, Hebei University of Technology, Tianjin, 300130 China; 3 Department of Respiratory and Critical Care Medicine, Tianjin Medical University General Hospital, Tianjin, 300052 China; 4 Institute of Solid Mechanics, Beihang University (BUAA), Beijing, 100191 China; 5 School of Electrical Engineering, Hebei University of Technology, Tianjin, 300130 China; 6 School of Architecture and Art Design, Hebei University of Technology, Tianjin, 300130 China; 7 Department of Engineering Science and Mechanics, The Pennsylvania State University, University Park, PA 16802 USA

**Keywords:** Environmental, health and safety issues, Electronic properties and materials

## Abstract

The accurate, continuous analysis of healthcare-relevant gases such as nitrogen oxides (NO_x_) in a humid environment remains elusive for low-cost, stretchable gas sensing devices. This study presents the design and demonstration of a moisture-resistant, stretchable NO_x_ gas sensor based on laser-induced graphene (LIG). Sandwiched between a soft elastomeric substrate and a moisture-resistant semipermeable encapsulant, the LIG sensing and electrode layer is first optimized by tuning laser processing parameters such as power, image density, and defocus distance. The gas sensor, using a needlelike LIG prepared with optimal laser processing parameters, exhibits a large response of 4.18‰ ppm^−1^ to NO and 6.66‰ ppm^−1^ to NO_2_, an ultralow detection limit of 8.3 ppb to NO and 4.0 ppb to NO_2_, fast response/recovery, and excellent selectivity. The design of a stretchable serpentine structure in the LIG electrode and strain isolation from the stiff island allows the gas sensor to be stretched by 30%. Combined with a moisture-resistant property against a relative humidity of 90%, the reported gas sensor has further been demonstrated to monitor the personal local environment during different times of the day and analyze human breath samples to classify patients with respiratory diseases from healthy volunteers. Moisture-resistant, stretchable NO_x_ gas sensors can expand the capability of wearable devices to detect biomarkers from humans and exposed environments for early disease diagnostics.

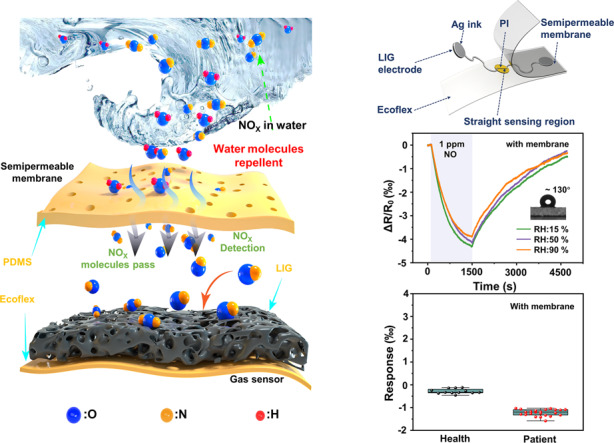

## Introduction

Wearable electronic devices can conform to the skin to capture mechanical^1^, thermal^2^, chemical^3^, electrical^4^, and biological signals^5,6^ for future health monitoring. The increasing interest in personal air quality monitoring and breath analysis has also spurred the demand for wearable gas sensors to accurately and continuously detect various health-relevant gases^7–9^. The nitrogen oxides (collectively termed NO_x_) generated from fuel combustion and petroleum refining are major atmospheric pollutants that lead to bronchitis and emphysema with heart-aggravating conditions^10–13^. Meanwhile, nitric oxide (NO), as an essential biomarker for airway inflammation, is of high interest for the noninvasive diagnosis and monitoring of respiratory diseases such as lung cancer and ventilator-associated pneumonia^14^. The NO concentration in the exhaled breath of asthma patients exceeds hundreds of parts per billion (ppb), whereas the level is less than several tens of ppb for healthy subjects^15–20^.

Studies on detecting NO_x_ have explored diverse sensitive nanomaterials such as graphene^21^, metal oxides^22^, conducting polymers^23^, and carbon nanotubes^24^. Compared with electrochemical cells, field-effect transistors, and other types of gas sensors, wearable graphene-based chemiresistive NO_x_ gas sensors from simple fabrication processes show low noise and high mechanical strength^25–31^. Unfortunately, graphene-based gas sensors without proper surface modification exhibit low sensitivity and poor selectivity due to few active sites and nonselective molecular adsorption^29–31^. The recently explored highly porous 3D laser-induced graphene (LIG) conveniently prepared by a fast, cost-effective, environmentally friendly laser scribing process^32–34^ supplies many active surface sites for gas-solid interactions for gas sensing^35,36^. In addition to exploring LIG with a high specific surface area and low contact resistance as a sensing electrode for detecting NO_2_ gas^37^, LIG can also be combined with other nanomaterials to form a P-N junction with increased carrier mobility to detect NO_2_^38,39^. Additionally, the changed thermal conductivity of pristine LIG in a vacuum environment allows for the detection of O_2_, N_2_, and CO_2_^36^. LIG-based NO_x_ gas sensors remain undeveloped.

As water molecules often occupy the active sites on the surface of sensing materials, the relative humidity (RH) often influences the adsorption and equilibrium processes of the target gas, resulting in large response fluctuations, especially in breath with an RH of 50–95%^40–43^. Attempts to mitigate the RH effect include the use of coated hydrophobic self-assembled monolayers (SAMs)^44,45^, coated moisture barrier layers^46^, integrated heating elements^47^, or electronic nose algorithms^48,49^. However, these methods usually increase the fabrication complexity and cost of the resulting sensing devices. Therefore, it is imperative to develop a facile strategy to design and fabricate moisture-resistant, stretchable NO_x_ gas sensors with a large response, rapid response/recovery rates, low limit of detection (LOD), and excellent selectivity to monitor local air quality and analyze breath for disease diagnostics.

Here, we design and demonstrate a moisture-resistant, stretchable LIG-based NO_x_ gas sensor, with the LIG sensing/electrode region sandwiched between semipermeable polydimethylsiloxane (PDMS) membrane and a soft elastomeric substrate. Tuning the laser processing parameters (e.g., laser power, image density, and defocus distance) yields a LIG with different morphologies (e.g., sheet, needle, closed rose petal, and collapsed hole-like microstructure), defect levels, and specific surface areas. A needlelike LIG with a large specific surface area of 296 m^2^/g and small defects of I_D_/I_G_ ≈ 0.46 is obtained with optimal laser processing parameters (i.e., power of 0.6 W, image density of 500 PPI, and defocus distance of 0 mm). The resulting sensor exhibits a large response of 6‰ (or 4‰), fast response/recovery of 134/388 s (or 113/296 s), and ultralow LOD of 4.0 ppb (or 8.3 ppb) to NO_2_ (or NO) at room temperature. Combining a high stretchability of 30% and a moisture-resistant property against an RH of 90%, the water-resistant, stretchable LIG-based gas sensing device has successfully detected outdoor air quality at different times of the day and analyzed the clinical breath samples to accurately classify patients with respiratory diseases from healthy human subjects.

## Results and discussion

### Fabrication of moisture-resistant, stretchable LIG-based gas sensors

**Fig. 1:**
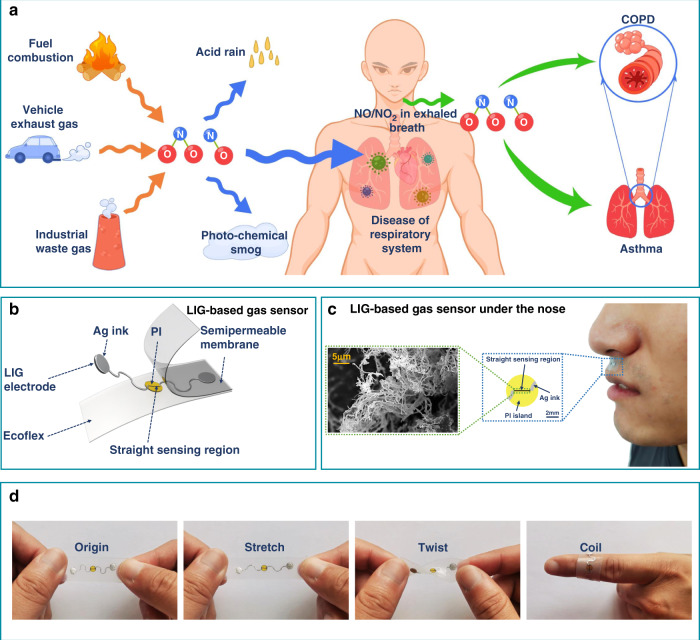
Moisture-resistant, stretchable gas sensing systems based on laser-induced graphene (LIG) foams for environmental monitoring and patient breath analysis. **a** Schematic illustrating NO_x_ (NO_2_ or NO)-related air pollution and the use of NO_x_ gas as a biomarker for representative human diseases. **b** Exploded view of the LIG-based moisture-resistant, stretchable gas sensor to show its structural layout. **c** Optical image of a representative LIG-based gas sensor attached below the nose of a human subject, with a zoomed-in view of the sensor and the scanning electron microscope (SEM) image of needlelike LIG shown in the inset. **d** Images of the stretchable LIG-based gas sensor before and after varying deformations (i.e., stretching, twisting, and coiling onto the finger)

Produced by fuel combustion, automobile exhaust, and industrial waste gas, NO_x_ is the primary pollutant in the atmosphere that causes bronchitis, emphysema, and other diseases after human inhalation (Fig. 1a). Exhaled NO_x_ is also an important biomarker for chronic obstructive pulmonary disease (COPD) and asthma^50–52^. The highly sensitive and selective, stretchable LIG-based gas sensor is designed to consist of a straight LIG sensing region and a serpentine Ag/LIG electrode on a soft elastomeric substrate (500 μm-thick Ecoflex) (Fig. 1b). The width in the LIG sensing region (150 μm) is much smaller than that of 0.5 mm in the electrode to provide much higher resistance and localized Joule heating in the sensing region. A thin semipermeable PDMS membrane with a thickness of 10 μm is applied to encapsulate the sensor to provide moisture-resistant properties. The rapid and low-cost fabrication process (Fig. S1) is also scalable, allowing rapid mass production for future commercialization (Fig. S2). Briefly, the commercial polyimide (PI) thin film attached to the glass slide is transiently heated by a commercial CO_2_ laser system in an ambient environment to form programmed 3D porous LIG patterns. The raster (or vector) mode is explored for the electrode (or sensing) region (Fig. S3). After transferring the LIG pattern onto the Ecoflex substrate, the serpentine electrode region is coated with Ag ink to further reduce the resistance in this region. Spin coating of a thin, gas-permeable PDMS film completes the preparation for the moisture-resistant, stretchable LIG-based gas sensor. The moisture-resistant LIG-based gas sensor can be conveniently attached to the skin below the nose for local environmental monitoring and breath analysis (Fig. 1c). The serpentine electrode and the strain isolation from the PI island underneath the sensing region (Fig. S4) provide the stretchable gas sensor with mechanical stability against various deformations, including stretching, twisting, and coiling on the finger (Fig. 1d).

### Optimization of laser processing parameters and characterization of LIG-based gas sensing response

**Fig. 2:**
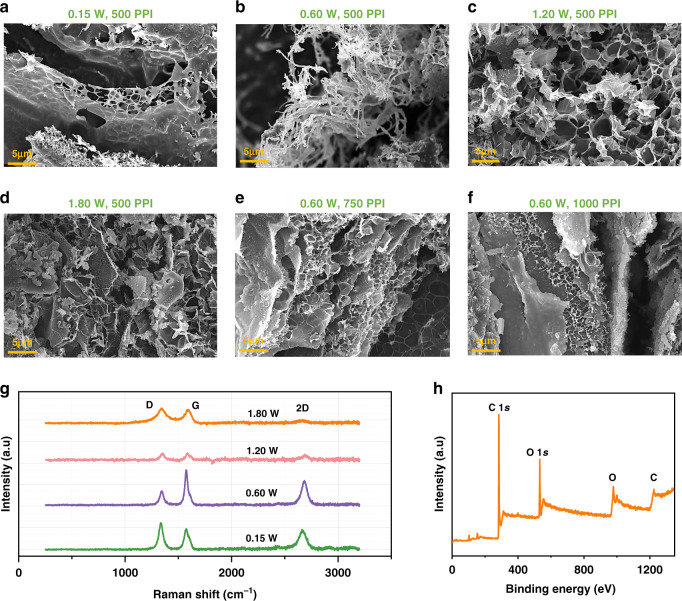
Characterizations of the LIG prepared with different laser processing parameters. **a**–**d** SEM images of the LIG prepared by laser powers of **a** 0.15 W, **b** 0.6 W, **c** 1.2 W, and **d** 1.8 W for a scanning speed of 2.54 mm/s and an image density of 500 PPI. SEM images of the LIG were prepared by the image density of **e** 750 PPI and **f** 1000 PPI for a laser power of 0.60 W and scanning speed of 2.54 mm/s. **g** Raman spectra of various LIG samples in **a**–**d**. **h** X-ray photoelectron spectroscopy (XPS) survey spectra of the LIG prepared by the optimal laser processing parameters (i.e., laser power of 0.6 W, image density of 500 PPI, and scanning speed of 2.54 mm/s)

The processing-structure relationship of the LIG is systematically investigated by varying key laser processing parameters, such as laser power and image density, while leaving other parameters unchanged (speed of 2.54 mm/s, defocus distance of 0 mm, vector mode) (Fig. 2). As the laser power is increased from 0.15 W to 0.6 W, the sheet-structured LIG (Fig. 2a) caused by the escape of local high-temperature gas changes into a needle-like morphology (Fig. 2b). Further increasing the power to 1.2 W and 1.8 W results in closed rose petals (Fig. 2c) and collapsed hole-like microstructures (Fig. 2d). The Raman spectra of these LIG structures (Fig. 2g) exhibit three predominant peaks: the D (~1350 cm^−1^), G (~1580 cm^−1^), and 2D (~2700 cm^−1^) peaks, confirming the presence of few-layered graphene. The smallest I_D_/I_G_ and largest I_2D_/I_G_ ratios of the needlelike LIG indicate its high degree of graphitization (Fig. S5 and Table S1)^53^. The smallest full width of half-maximum (FWHM) of the G peak from the needlelike LIG prepared with a power of 0.6 W also indicates high-quality graphene (Table S1). After determining the optimal laser power of 0.6 W, the image density that is controlled by the pulse per inch (PPI) is further investigated to modulate the LIG morphology (Fig. S6). For a constant laser power of 0.6 W, the increase in the image density from 500 PPI to 750 PPI and then to 1000 PPI leads to overheating and the destruction of the needle-like structure (Fig. 2b, e-f). The increase in the image density also leads to increased defects and decreased quality in the LIG, as observed in the increased I_D_/I_G_ ratio (Fig. S7 and Fig. S8) and FWHM of the G peak (Table S2). This result is possibly due to the increased overlap between the laser paths^34^. By controlling the laser spot size and energy, the defocus distance is another commonly used processing parameter to modulate the LIG morphology (Fig. S9). As the defocus distance is increased from 0 to 9 mm, the I_D_/I_G_ ratios from the Raman spectra of the resulting LIG increase (Fig. S10) and indicate increased defects in the LIG (Fig. S11 and Table S3). The increased defects can be attributed to insufficient energy and thus a low target temperature. X-ray photoelectron spectroscopy (XPS) of the LIG prepared with the optimal parameters revealed clear carbon (C) and oxygen (O) features (Fig. 2h). The deconvoluted C1 s into C–C (284.5 eV), C–O (285.2 eV), and C=O (288.7 eV) (Fig. S12) is also consistent with a previously reported result^54^.

**Fig. 3:**
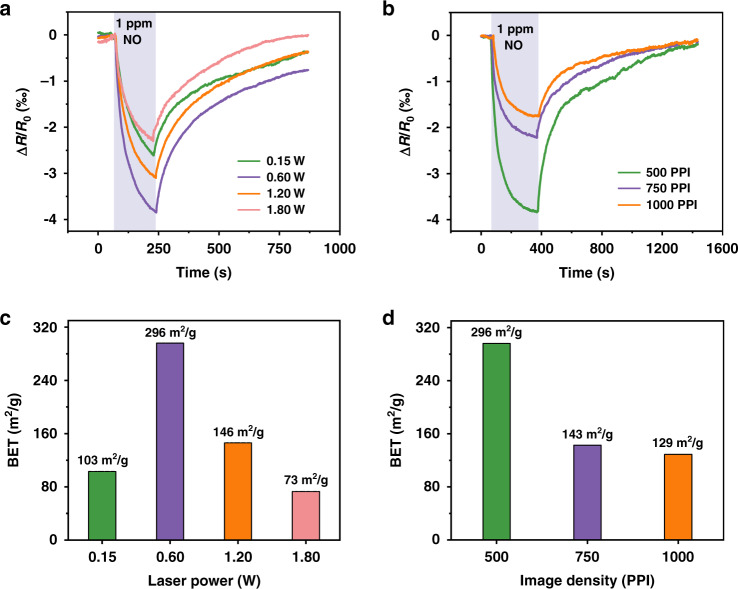
Effects of laser processing parameters on gas sensing performance. Sensing performance of the LIG-based gas sensors prepared with different **a** powers and **b** image densities to 1 ppm NO gas, with their specific surface areas shown in **c** and **d**

With *R*_*0*_ and *R* denoting the steady resistance in air and the target gas, the gas sensor response is defined as *ΔR/R*_*0*_ = *(R−R*_*0*_*)/R*_*0*_. At a room temperature of 25°C (bias voltage of 0.05 V), the p-type LIG-based chemiresistive gas sensor reduces its resistance (Fig. 3a, b) upon exposure to oxidizing NO_x_ (electron acceptor). As the laser power is increased from 0.15 W to 0.6 W, 1.2 W, and 1.8 W, the response of the LIG-based gas sensor first increases from 2.60 to 4.0‰ but then decreases to 3.10‰ and then 2.20‰ (to 1 ppm NO) (Fig. 3a). The highest response from the needlelike LIG-based gas sensor prepared at a power of 0.6 W is likely attributed to its largest specific surface area, as confirmed by the Brunauer-Emmett-Teller (BET) measurements **(**Fig. 3c). In the investigation of the effect of the image density on the gas sensor response, the increase in the image density from 500 to 1000 PPI shows a decreased response from 4.0 to 1.7‰ (Fig. 3b), which is also likely due to the decreased specific surface area from 296 to 129 m^2^/g (Fig. 3d). For the effect of the defocus distance, its increase from 0 to 9 mm results in a decreased gas sensor response from 4.1‰ to 0.9‰ (Fig. S13). It is interesting to note that the response of the gas sensor prepared by a defocus distance of 2 mm is roughly the same (~4‰) as that from 0 mm. This is attributed to the similar LIG morphology created by the similar energy levels, as the defocus distance of 2 mm is less than the focal depth (2.54 mm) of the laser lens (Fig. S9). Therefore, the optimal laser processing parameters with a power of 0.6 W, image density of 500 PPI, and defocus distance of 0 mm are chosen in the following studies unless otherwise specified.

### Sensing performance characterization of LIG-based gas sensors for NO_x_

**Fig. 4:**
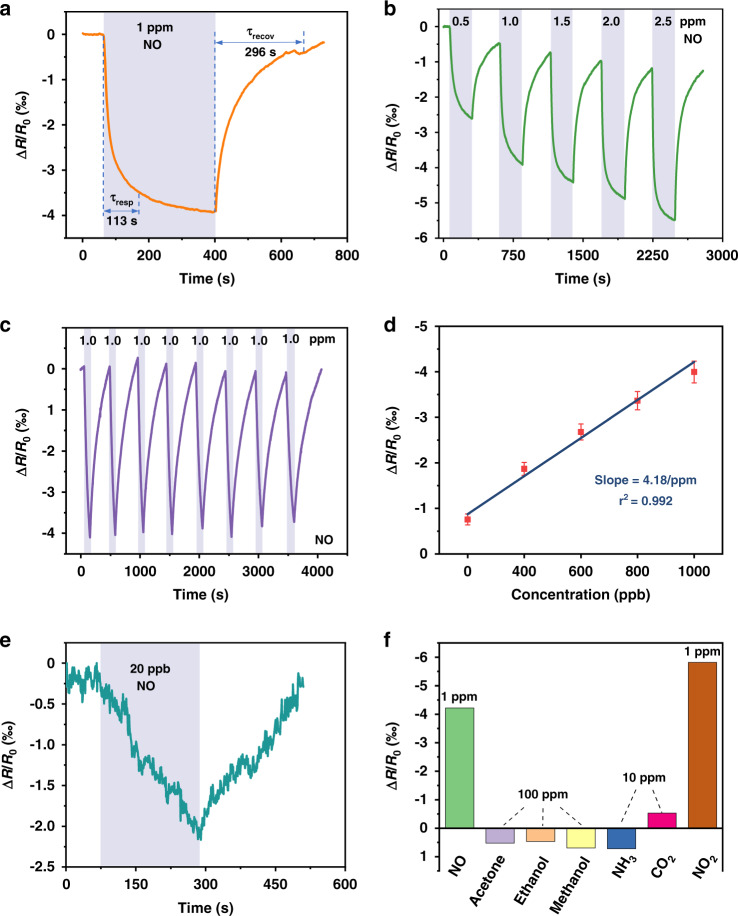
Gas sensing performance evaluation of the LIG-based gas sensor prepared with the optimal laser processing parameters. **a** Typical response curve of the gas sensor to 1 ppm NO. **b** Dynamic response curves of the gas sensor to NO with concentrations increasing from 0.5 to 2.5 ppm. **c** Repeatability test of the gas sensor to 1 ppm NO for eight consecutive cycles. **d** Calibration curve with a linear fit obtained from the sensor response to NO from 200 to 1000 ppb. **e** Experimental demonstration of the sensor response to 20 ppb NO. **f** Selectivity test of the gas sensor to NO_x_ over other interfering gases

The LIG-based gas sensor exhibits a fast response/recovery (113/296 s) to 1 ppm NO even at room temperature (Fig. 4a). The continuous response curve to NO with progressively increasing concentration from 0.5 to 2.5 ppm exhibits the increased response from 2.6 to 5.5‰ (Fig. 4b), indicating excellent dynamic response/recovery at room temperature. The incomplete recovery in the dynamic response is attributed to the short recovery time used in the testing and the residual charge carrier on the LIG repeatedly exposed to NO_x_^55,56^. However, the elevated working temperature can be used to enhance the desorption process of the absorbed gas molecules to reduce incomplete recovery. For instance, the recovery time decreases from 400 to 210 s as the operating temperature is increased from 25 to 60°C (Fig. S14). The elevated temperature can be easily achieved from self-heating (Joule heating), which comes from the significantly increased resistance of the sensing region compared to that of the electrode. As the applied voltage is stepwise increased from 0 to 15 V to raise the temperature to 65°C, the resistance of the LIG gas sensing platform only gradually decreases by 3.1% (Fig. S15a), consistent with the literature report^38^ attributable to the negative temperature coefficient^57^. The small change in resistance has a negligible effect on the input power for heating (Fig. S15b). Although the operating temperature of 60°C is higher than the desired temperature on the skin, it is possible to exploit a heat sink or thermal isolation layer to significantly reduce the temperature at the sensor/skin interface to avoid the adverse thermal effect on the skin surface^58,59^. The relatively stable response of ~4.0‰ and fast response/recovery (110/330 s) from the gas sensor to 1 ppm NO over eight consecutive cycles indicate excellent repeatability and reversibility (Fig. 4c), which is crucial for practical gas sensing. The long-term stability of the sensor is also confirmed by the almost unchanged response to 1 ppm NO over 15 days (Fig. S16). Because NO_2_ is more oxidizing and has better electron-withdrawal properties than NO, the LIG-based gas sensor exhibits a similar but more pronounced response when exposed to NO_2_ (Fig. S17).

The linear fit of the sensor response to the NO gas concentration from 200 to 1000 ppb yields a slope of 4.18 ppm^−1^ with a correlation coefficient (*R*^*2*^) of 0.992 through the least square fit^60^ for the calculation of the theoretical limit of detection (LOD) (Fig. 4d and Fig. S18). As the LOD is defined as 3 × RMS_*noise*_/Slope^61^ with RMS_*noise*_ to be the standard deviation in the baseline of the response curve, the LOD to NO is calculated to be 8.3 ppb. In practical applications, the signal-to-noise ratio (SNR), defined as ∆R/RMS_*noise*_, determines the actual LOD that approximately corresponds to the signal with SNR = 3. Although the sensor response to NO_x_ is relatively small (e.g., 4‰ to 1 ppm NO and 6‰ to 1 ppm NO_2_), the SNR values of 463 to NO and 679 to NO_2_ are still much larger than those of the other 2D nanomaterial-based gas sensors^62^. The highly porous LIG with a high specific area provides low contact resistance to give low noise and high SNR. The validation of the actual LOD can be challenging with the static gas testing setup in this work, but the response curve to 20 ppb NO still exhibits a steady response of 2.2‱ with an SNR of 42.7, as well as rapid response/recovery (Fig. 4e). The LOD of the LIG-based gas sensor is sufficient for air quality monitoring and breath analysis, as the early warning range of NO_2_ in the ambient atmosphere is 0.6–5 ppm^63,64^ and the exhaled NO from patients with asthma and halitosis is >100 ppb^65^. Furthermore, the response of the LIG-based gas sensor to NO_x_ (1 ppm of NO or NO_2_) is significantly higher than that of other interfering gases (e.g., 10 ppm of NH_3_ and CO_2_, 100 ppm of acetone, ethanol, and methanol). When the P-type chemiresistive LIG gas sensor is exposed to an oxidizing NO_x_ gas, the electrons in the valence band of the LIG are extracted by the adsorbed NO_x_, forming the hole (main carriers) accumulation zone. The lowest unoccupied molecular orbital (LUMO) determines the number of transferred electrons. As the LUMO of NO_x_ gas molecules is lower than that of other gases^66^, more electrons are transferred from the LIG to give a larger response. Therefore, the LIG exhibits excellent selectivity to NO_x_ (Fig. 4f). In a representative comparison, although the concentration of CO_2_ is increased from 10 to 100 ppm, the response only increases from 0.52‰ to 0.96‰ (from one-eleventh to one-sixth of the response to 1 ppm NO_x_) (Fig. S19). Therefore, it is still possible to distinguish low-concentration NO_x_ from high-concentration CO_2_ and other interfering gases that exhibit reducing characteristics^67^. While it is challenging to further distinguish NO and NO_2_, the response/recovery time and magnitude of the response can be used.

### Gas sensing mechanism of LIG-based sensors

**Fig. 5:**
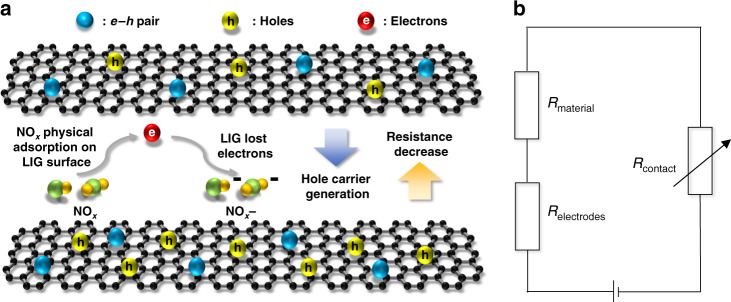
Sensing mechanism and equivalent circuit diagram of the LIG-based gas sensor. **a** Sensing mechanism of the chemiresistive LIG-based gas sensor for NO_x_. **b** Equivalent circuit diagram of the gas sensor

The p-type LIG-based chemiresistive gas sensor^68,69^ reduces its resistance upon exposure to oxidizing NO_x_ (electron acceptor)^70^. This resistance decrease is mediated by the direct charge transfer on the surface, where the electrons in the valence band of the LIG are extracted by adsorbed nitrogen oxides^37^, i.e., *NO*_*x*_ *(gas)* + *e*^−^ *↔ NO*_*x*_^−^ (*ads*). The NO_x_ adsorbed on the LIG surface continuously extracts electrons and extends the hole (main carriers) accumulation zone on the LIG surface to lower the resistance (Fig. 5a). The total resistance of the as-prepared gas sensor is the sum of the contact resistance between LIG and NO_x_ gas, the resistance of the intrinsically sensitive nanomaterial, and the resistance of the electrodes, i.e., *R*_*total*_ = *R*_*contact*_ + *R*_*material*_ + *R*_*electrodes*_ (Fig. 5b). As *R*_*electrodes*_ is often negligible, the change in *R*_*total*_ of the LIG sensor during NO_x_ adsorption/desorption can be attributed to the modulation of sorption sites.

### Demonstration of moisture resistance and stretchability of the LIG-based gas sensor for practical applications

**Fig. 6:**
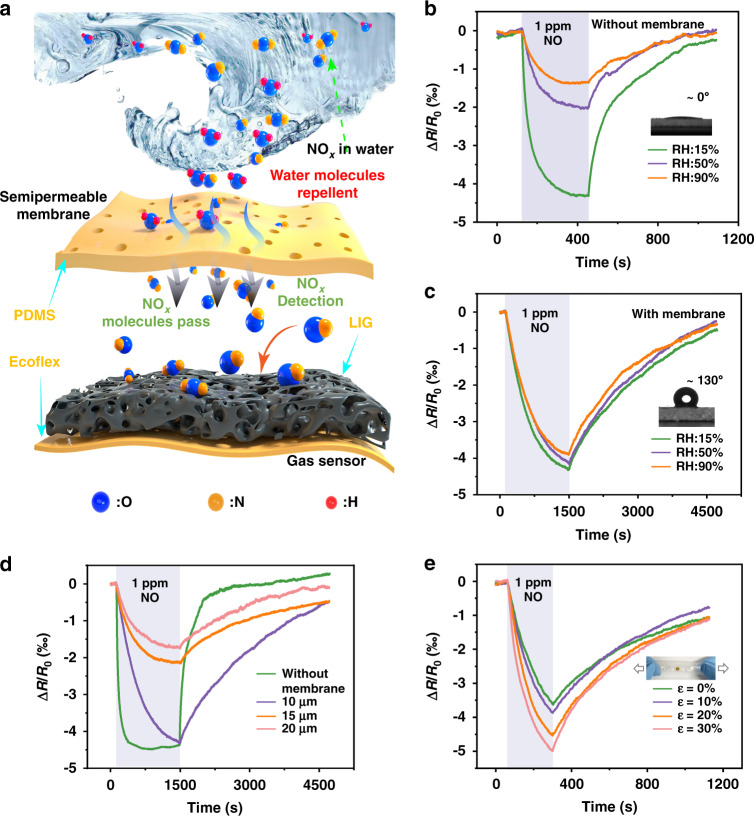
The moisture-resistant and stretchable performance of LIG-based gas sensor. **a** Schematic illustration showing humidity-free gas sensing under wet conditions. Response of gas sensors in different relative humidity environments **b** without and **c** with a semipermeable PDMS membrane, with the water contact angle shown in the inset. **d** Thickness effect (10–20 μm) of the semipermeable membrane on the gas sensing response. **e** Response of the LIG-based gas sensor to 1 ppm NO upon stretching from 0 to 30%

The high RH of 89–97% in exhaled breath^65^ poses a significant challenge for gas sensing, as it often drastically affects the response due to the adsorption of water molecules on the active sensitive layer, especially hydrophilic LIG (water contact angle of ~0°). The semipermeable PDMS membrane coated on the LIG sensing region could provide an effective diffusion pathway for NO_x_ through the siloxane backbone (Si-O)^71^ while repelling water and aqueous components due to the reduced surface energy from the methyl group (Si-CH_3_) (Fig. 6a). The comparison in the response of the LIG-based gas sensor without (Fig. 6b) or with (Fig. 6c) the semipermeable PDMS membrane to 1 ppm NO for RH from 15 to 90% clearly reveals the role and effect of water resistance. In comparison to the sensor without PDMS, which shows a drastic decrease from 4 to 1.3‰ as the RH increases from 15 to 90% (Fig. 6b), the sensor with hydrophobic PDMS (water contact angle of ~130°) exhibits almost negligible changes (Fig. 6c). The use of the semipermeable PDMS membrane, however, increases the response/recovery time from 130/350 s to 890/2810 s due to the decreased diffusion rate of the gas molecules through PDMS^72^. This issue can be mediated at an elevated operating temperature from self-heating. For instance, the response/recovery time decreases from 890/2810 s to 403/591 s (with small effects on the magnitude of the response), as the operating temperature is increased from 25 to 60°C (Fig. S20). Moreover, the gas sensor with a semipermeable membrane also exhibits good dynamic response/recovery at different operating temperatures (Fig. S21). A thinner PDMS layer can also be desirable to reduce the diffusion time. Meanwhile, the gas sensor with the thinnest PDMS membrane of 10 μm can maintain the large response of ~4‰ as that without PDMS (Fig. 6d).

Benefitting from the stretchable serpentine electrode and the strain isolation, the gas sensor is capable of withstanding a uniaxial tensile strain of 30% (Fig. S22), which is sufficient to accommodate the maximum skin deformation^73^. Compared with the U-shaped electrode that shows 0.7‰ resistance fluctuation for 30% stretching, the serpentine electrode provides a much lower fluctuation of 0.3‰ (Fig. S23), confirming the advantage of choosing the serpentine design. As the uniaxial strain is increased from 0 to 30%, the response of the LIG-based gas sensor to 1 ppm NO slightly increases from 3.5 to 5.0‰ (Fig. 6e), which is likely attributed to the locally torn graphene surface upon stretching^74^. As a result, the LIG-based gas sensor with an ultralow LOD (8.3 and 4.0 ppb to NO and NO_2_), fast response/recovery (113/296 and 134/388 s to 1 ppm NO and NO_2_), high stretchability (30%), and moisture-resistant property compares favorably with other stretchable gas sensors based on varying nanomaterials (Table S4).

### Demonstrations of the LIG-based gas sensor for environmental monitoring and breath analysis

**Fig. 7:**
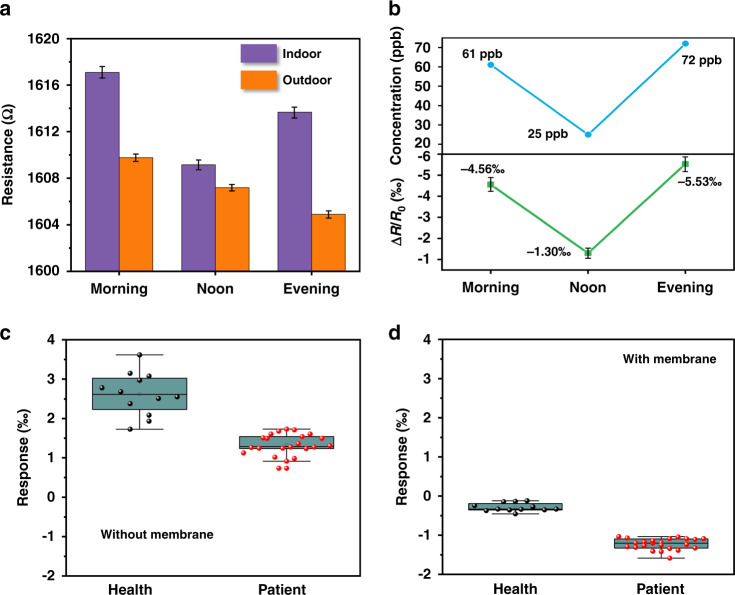
Demonstration of the LIG-based gas sensor for environment monitoring and breath analysis. **a** Resistance variations of the LIG-based gas sensor to monitor outdoor air at different times of the day (i.e., morning, noon, and evening). **b** Comparison between the actual NO_2_ concentrations in the environment (top) and the response from the gas sensor (bottom). Response of the gas sensor **c** without and **d** with the semipermeable membrane to human exhaled breath samples from patients with respiratory diseases and healthy volunteers

The applications of the LIG-based gas sensor to monitor the outdoor environment at different times of the day (e.g., morning, noon, and evening) allow air quality monitoring of NO_2_ from car exhaust (Fig. 7a, b). The sensor is first placed inside a closed gas tank filled with indoor ambient air for 120 s. Next, the sensor is moved to an outdoor environment for 360 s before it is placed back into the closed gas tank. The detected responses in the morning and evening of −4.56‰ and −5.53‰, respectively, are larger in magnitude than those of −1.30‰ at noon (Fig. 7b). Assuming that NO_2_ is the major active gas molecule, the use of the linear fit from Fig. S17e provides the estimated concentration of 769/280/914 ppb at morning/noon/evening, which is consistent with the trend captured by the commercial NO_2_ gas sensor (TB200B, ECsense) (Fig. 7b). More importantly, the LIG-based gas sensor can be applied to analyze human breath samples for potential diagnostic confirmation of respiratory diseases such as asthma or COPD. In the representative demonstration, human breath samples collected from 23 patients with asthma or COPD and 12 healthy volunteers were analyzed (Table S5). To provide high operation reliability, exhaled breath samples from healthy volunteers and patients with respiratory diseases are first collected into an aluminum foil gas collecting bag^75,76^ and then injected into a closed chamber for static detection (Fig. S24 and Fig. S25). Real-time detection and breath analysis will be pursued in future studies. The response values from the gas sensor without the semipermeable PDMS membrane are quite scattered (in the range from 1.73–3.61‰) even for healthy volunteers, likely due to the RH effect^77^ (Fig. 7c). The adsorption of water molecules on the active sensitive layer causes the sensor to exhibit a positive response (Fig. S26 and Fig. S27a). In contrast, the sensor with the semipermeable membrane can effectively filter out the humidity effect to provide the response with a much smaller variation in the range from −0.12 to −0.46‰ (Fig. 7d and Fig. S27b). Meanwhile, the classification accuracy is significantly improved, as the sensor response values from the respiratory disease patients are approximately 4.8-fold greater than those of healthy volunteers (Fig. 7d).

## Conclusion

In summary, we have reported the design, fabrication, and demonstration of a highly sensitive, moisture-resistant, and stretchable LIG-based gas sensor for environmental monitoring and patient breath analysis. Controlled by the laser processing parameters (e.g., laser power, image density, and defocus distance), the laser direct writing process can yield LIG sensing regions with different morphologies. Due to its large specific surface area of 296 m^2^/g, the needlelike LIG prepared with the optimal parameters (power of 0.6 W, image density of 500 PPI, and defocus of 0 mm) exhibits the largest response of 4‰, fast response/recovery of 113/296 s, and ultrahigh SNR of 463 to 1 ppm NO at room temperature. The serpentine LIG electrode and strain isolation from the PI island further allow the gas sensor to withstand a uniaxial tensile strain of 30% to accommodate skin deformation and motions. Taken together with the ultralow LOD of 8.3 ppb and the water-resistant property of the semipermeable PDMS membrane, the NO_x_ gas sensor can conveniently and quickly monitor air quality and analyze breath samples to classify patients with asthma/COPD from healthy volunteers.

## Experimental section

### Fabrication of the LIG-based NO_x_ gas sensor

The thin PI film (50 μm, Gold Finger, China) laminated on a water-soluble tape (25 mm wide, Yong Ri, China) was first attached to a glass slide by double-sided tape. Next, the LIG serpentine electrode (Fig. S28, black) was formed on the PI surface by photothermal ablation with a CO_2_ laser (Universal Laser Systems ULS 2.30, power *P*_max_ = 30 W, wavelength of 10.6 μm) in raster scanning mode (power of 3 W, speed of 127 mm/s, image density of 500 PPI, and defocus distance of 0 mm). The LIG sensing region (Fig. S28, green) was also formed by the same laser scribing process but in vector mode at a power of 0.6 W and a speed of 2.54 mm/s. The cutting of the programmed LIG pattern (Fig. S28, red) was achieved by the same laser system at a power of 30 W and a speed of 152 mm/s. The resulting sample was immersed in water to dissolve the water-soluble tape and release the LIG pattern from the glass substrate. After cleaning the LIG surface with ethanol and water, gentle stirring removed dust and contaminants. After the cleaned LIG adhered to another water-soluble tape, a 500 μm-thick Ecoflex (Ecoflex 00-30, Smooth-on) layer was cast on the back of the PI surface and cured at room temperature for 24 hours. The water-soluble tape exposed the LIG sensing region for spin coating of the PDMS thin film and the electrode region for coating and sintering of Ag ink (8821X, Shenggelu Technology, China), completing the gas sensor fabrication.

### Characterization

Characterizations were carried out directly on LIG films unless otherwise specified. SEM images were collected by a field emission scanning electron microscope (JSM 7100 F, JEOL). XPS and Raman spectra were recorded using an ESCALAB 250 photoelectron spectrometer (ESCALAB 250Xi, Thermo Fisher Scientific) and a laser micro Raman spectrometer (inVia Reflex, Renishaw), respectively. BET analysis of the LIG powder scratched from LIG films was carried out by a specific surface area and porosity analyzer (ASAP2020M + C, Micromeritics). Contact angle images were obtained by a fully automatic contact angle measuring system (DSAHT, KRUSS GmbH).

### Calculation of the Concentration of the VOC

The concentration of the VOC was obtained by injecting the required quantity of anhydrous liquid analytes into a sealed glass container using a microliter syringe. The concentration (*C* in ppm) of the VOC in the chamber was calculated using the following equation^78,79^:

$$C = \frac{{22.4\rho TV_S}}{{273MV}} \times 1000,$$

where *ρ*, *V*_*S*_, and *M* are the density (g ml^−1^), volume (μL), and molecular weight (g mol^−1^) of the anhydrous liquid VOC, *T* is the testing temperature (K), and *V* is the volume of the glass container (L) filled with the VOC.

### Collection of breath samples

Human breath samples were collected from patients with respiratory diseases at the Tianjin Medical University General Hospital and healthy volunteers at the State Key Laboratory of Reliability and Intelligence of Electrical Equipment (statistics provided in Table S5).

### Testing of gas sensors

The response of the gas sensors was measured and recorded by a source meter (Keithley 2400) at a constant voltage of 0.05 V unless otherwise specified. Different concentrations of NO_x_ were prepared by diluting the commercial calibration gas of 100 ppm NO_x_ and fully mixing it with air in the chamber (volume of 10 L). The different relative humidities in the chamber were prepared by the saturated salt solution method.

## Supplementary information

Supplementary Information

## Data Availability

The datasets generated during and analyzed during the current study are available from the corresponding author on reasonable request.
