# Supplementary material for: Moisture-resistant, stretchable NOx gas sensors based on laser-induced graphene for environmental monitoring and breath analysis
Source: Microsyst Nanoeng. 2022 Jul 8;8:78. doi: 10.1038/s41378-022-00414-x (PMC9270215; doi:10.1038/s41378-022-00414-x)
Supplement: Supplementary file 1 — Supplementary Information [file 41378_2022_414_MOESM1_ESM.docx]

Supporting Information

Moisture-resistant, stretchable NO_x_ gas sensors based on laser-induced graphene for environmental monitoring and breath analysis

*Li Yang^*, †^, Guanghao Zheng^††^, Yaoqian Cao^¶^,* *Chuizhou Meng^††^, Yuhang Li*^┴^*, Huadong Ji^††^, Xue Chen^‡‡^, Guangyu Niu^§§^, Jiayi Yan^††^, Ye Xue^†^, Huanyu Cheng^*, ‡^.*

^†^State Key Laboratory of Reliability and Intelligence of Electrical Equipment, School of Health Sciences and Biomedical Engineering, Hebei University of Technology, Tianjin, 300130, China

^††^School of Mechanical Engineering, Hebei University of Technology, Tianjin, 300130, China

^¶^Department of Respiratory and Critical Care Medicine, Tianjin Medical University General Hospital, Tianjin, 300052, China

^┴^Institute of Solid Mechanics, Beihang University (BUAA), Beijing, 100191, China

*^‡‡^*School of Electrical Engineering, Hebei University of Technology, Tianjin, 300130, China

^§§^School of Architecture and Art Design, Hebei University of Technology, Tianjin, 300130, China

^‡^Department of Engineering Science and Mechanics, The Pennsylvania State University, University Park, PA, 16802, USA

Corresponding Author

*Email: [yangli5781@126.com](mailto:yangli5781@126.com).

*Email: [huanyu.cheng@psu.edu](mailto:huanyu.cheng@psu.edu).


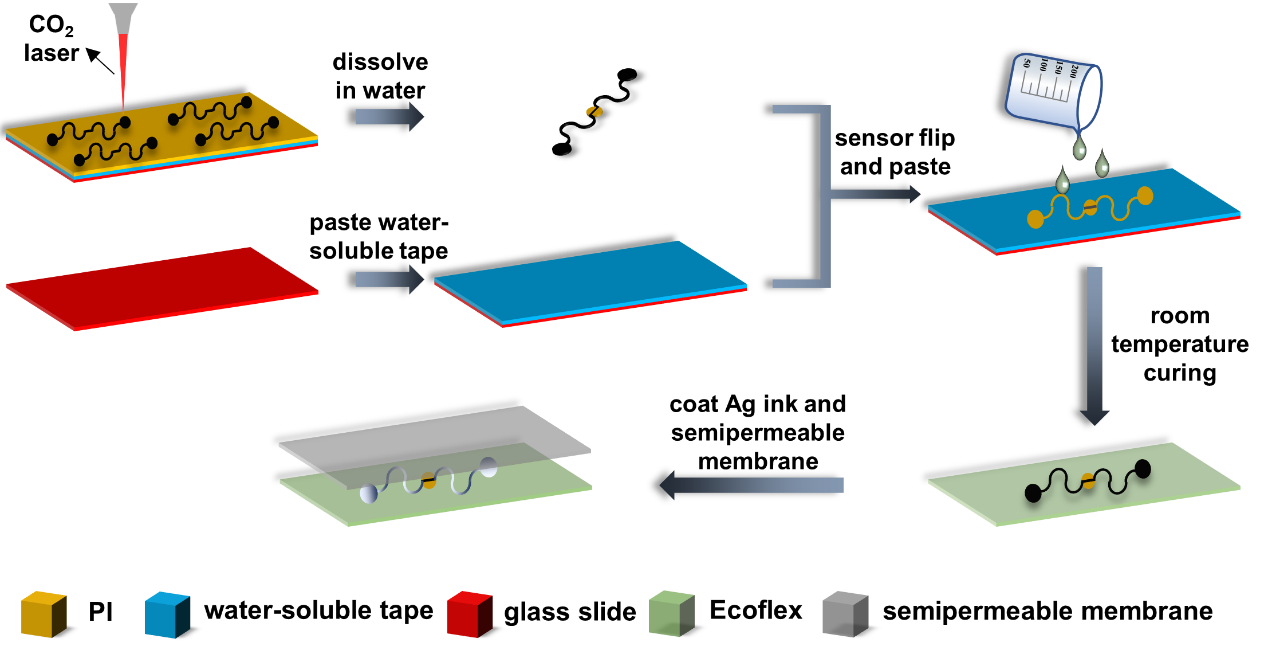


**Figure S1.** Schematic showing the fabrication process of the water-resistant, stretchable LIG-based gas sensor.


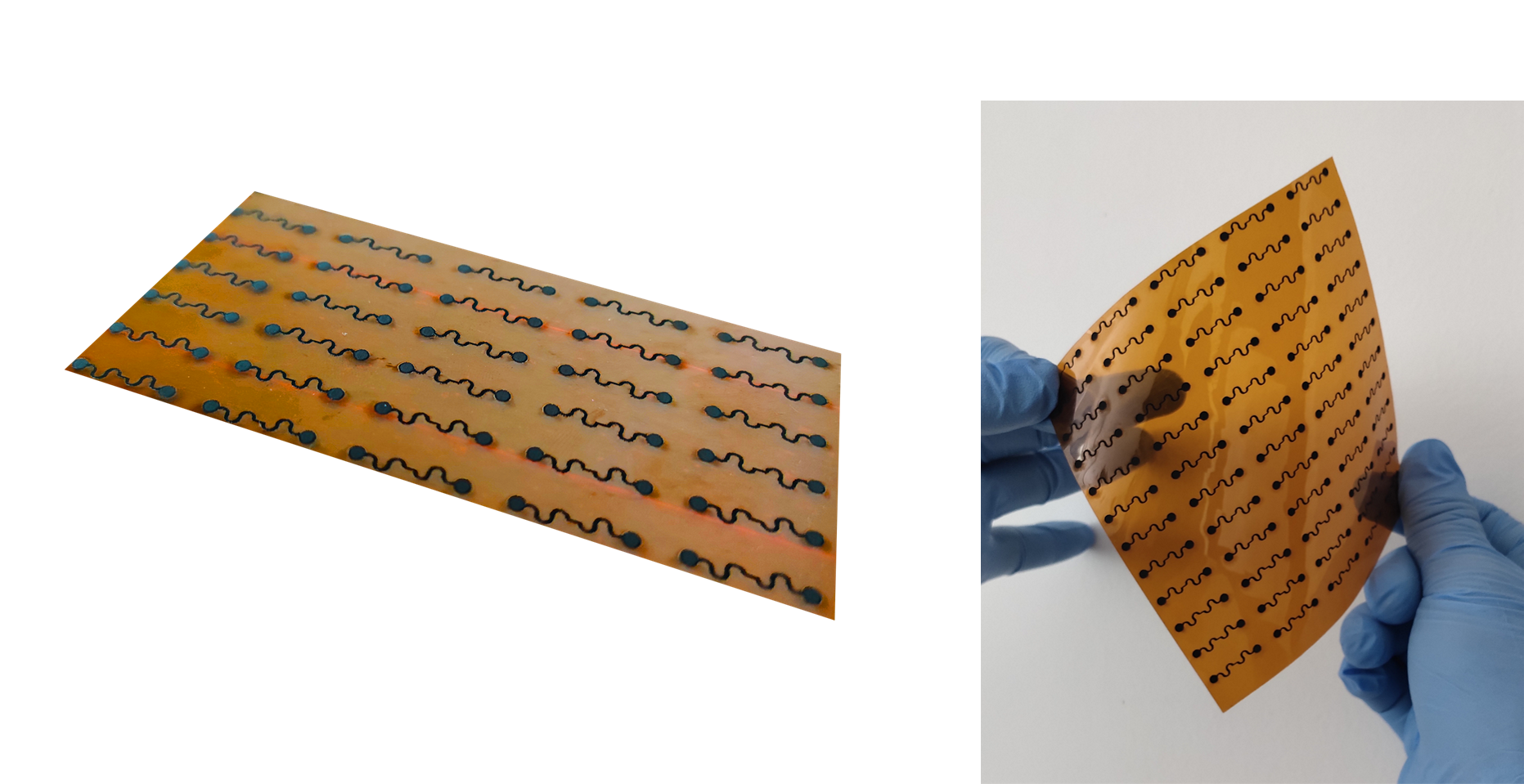


**Figure S2.** Demonstration of the low-cost, scalable manufacturing of the stretchable LIG-based gas sensor for potential mass production.


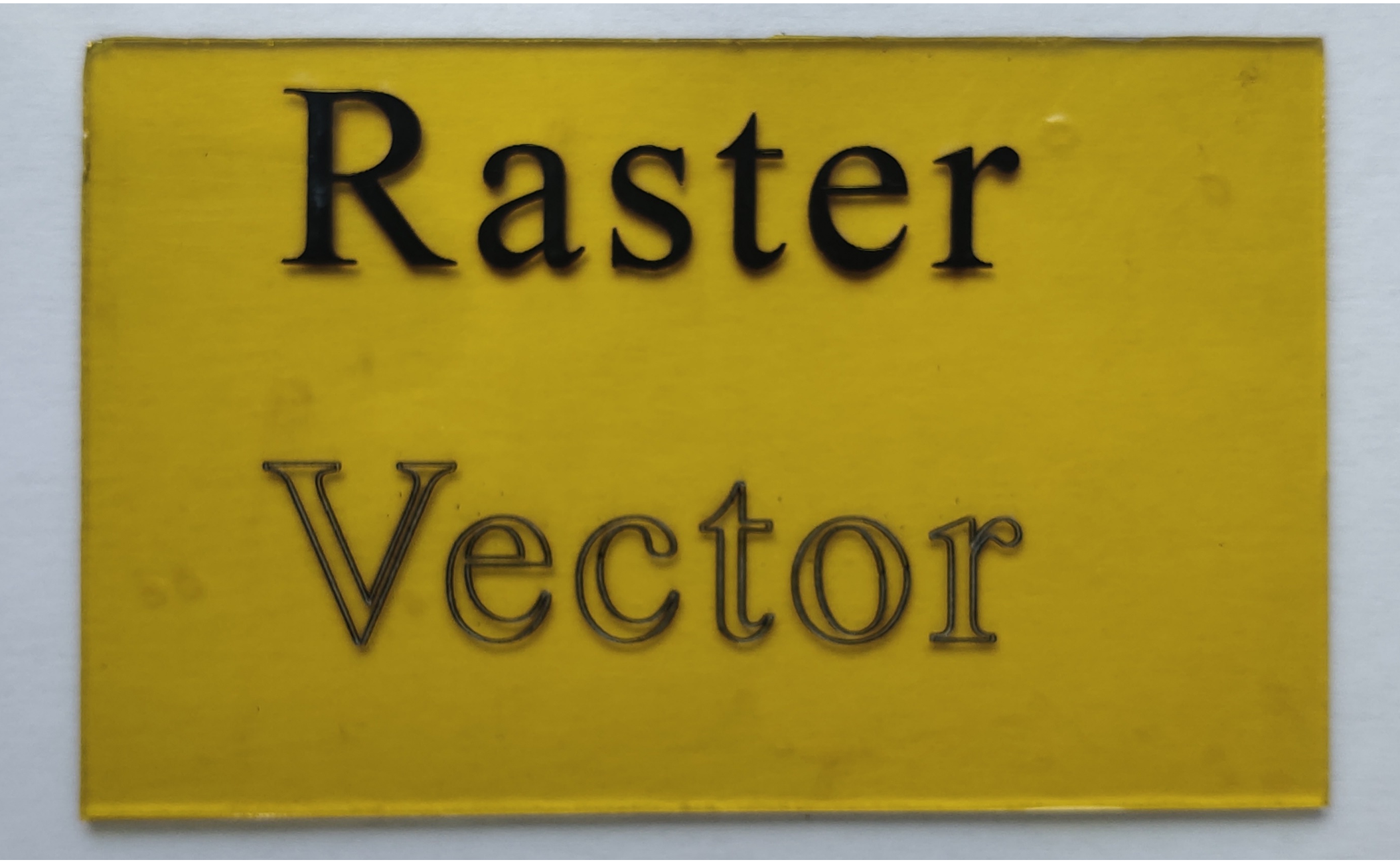


**Figure S3.** Optical image of the LIG patterns prepared by the raster (top) and vector (bottom) modes.


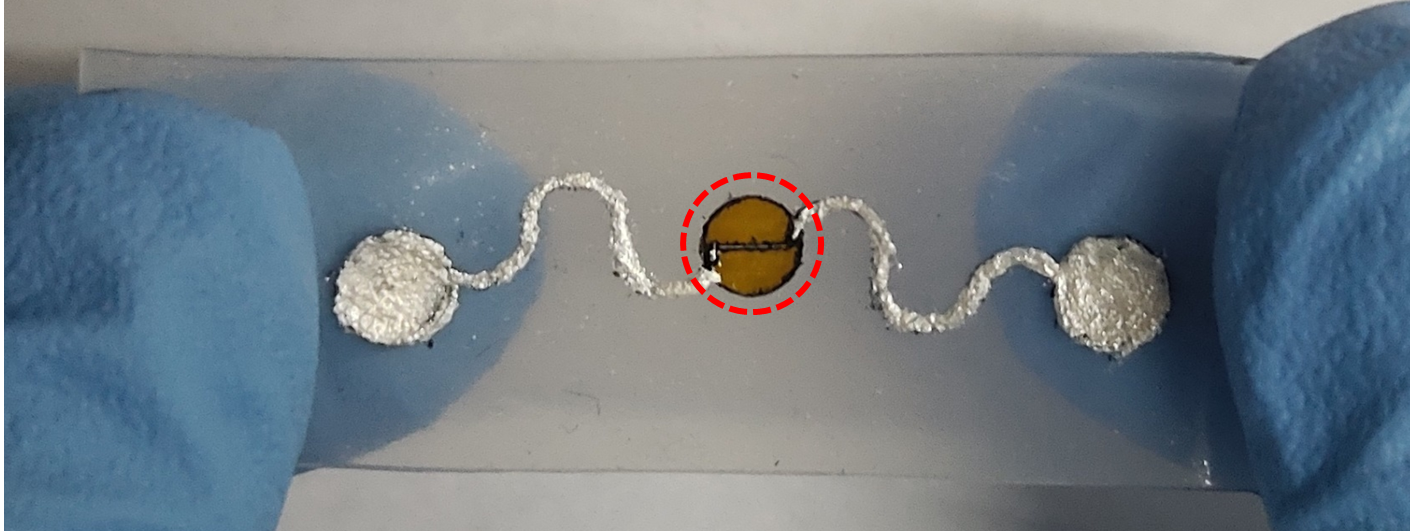


**Figure S4.** Optical image of the stretchable gas sensor with the PI island beneath the sensing LIG for improved strain isolation effect highlighted in the red circle.


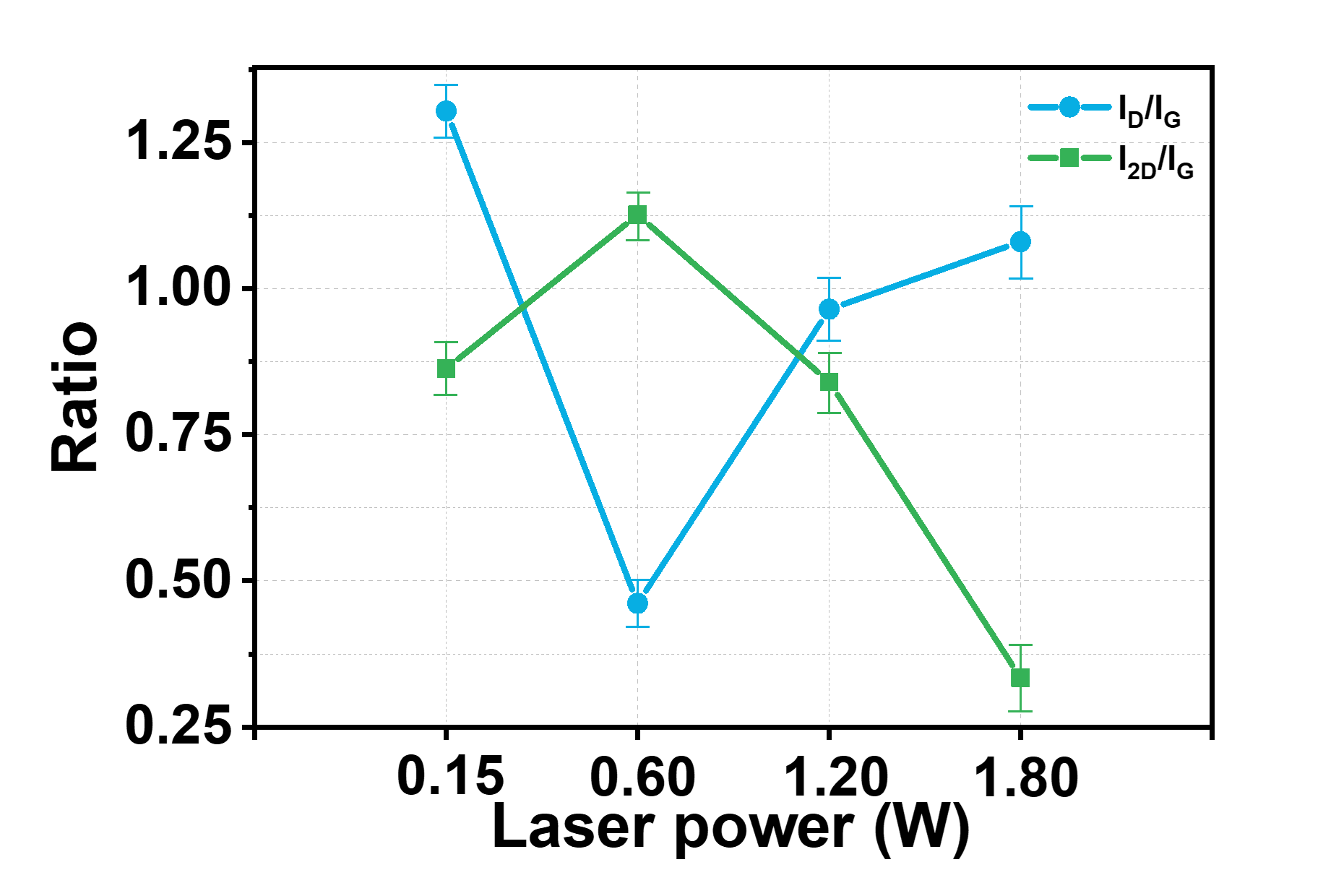


**Figure S5.** Analysis of the peak intensity ratios of the D and 2D to G obtained from the Raman spectra of the LIG prepared by varying laser powers.


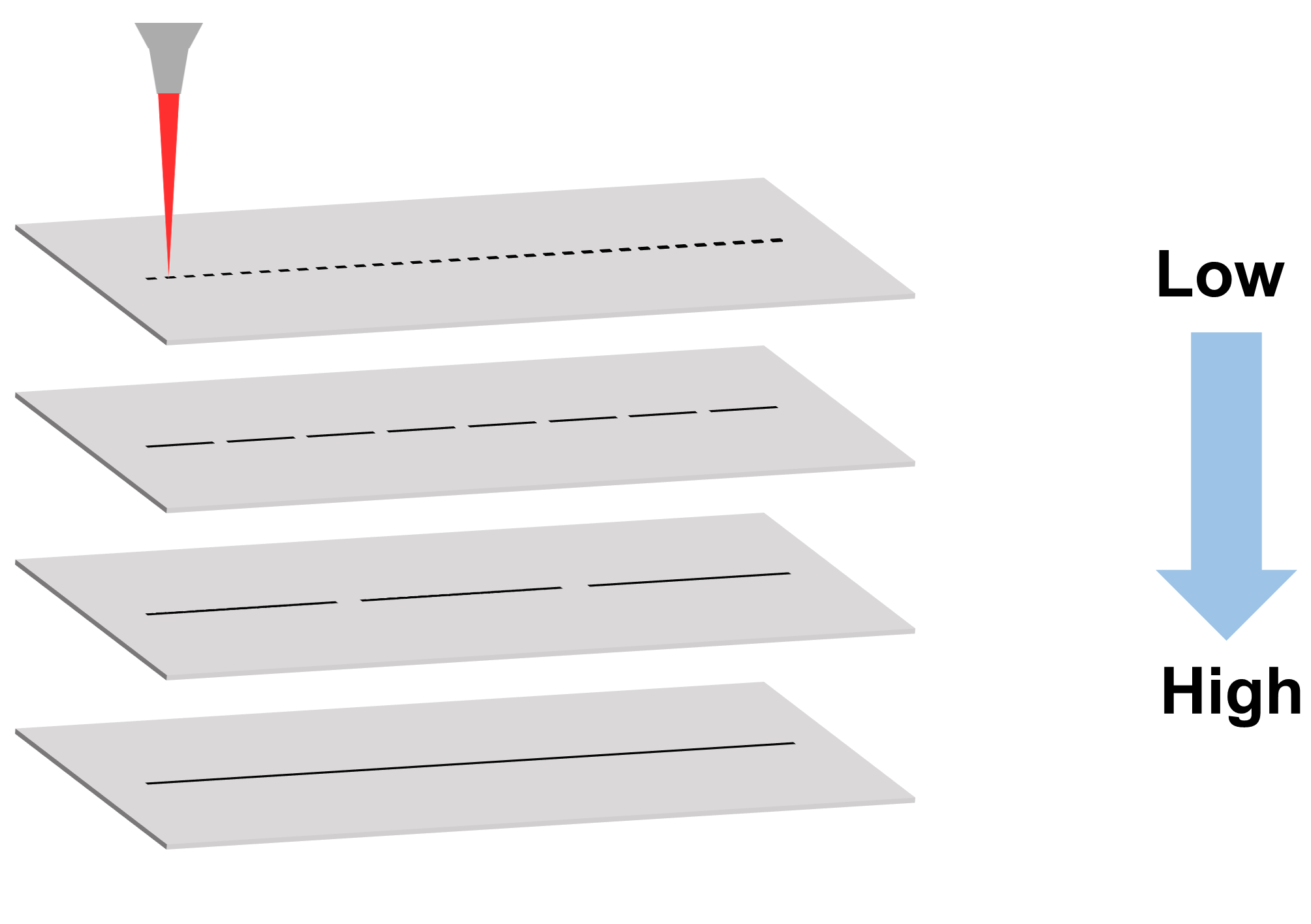


**Figure S6.** Schematic to show the LIG pattern formed by the increasing image density.


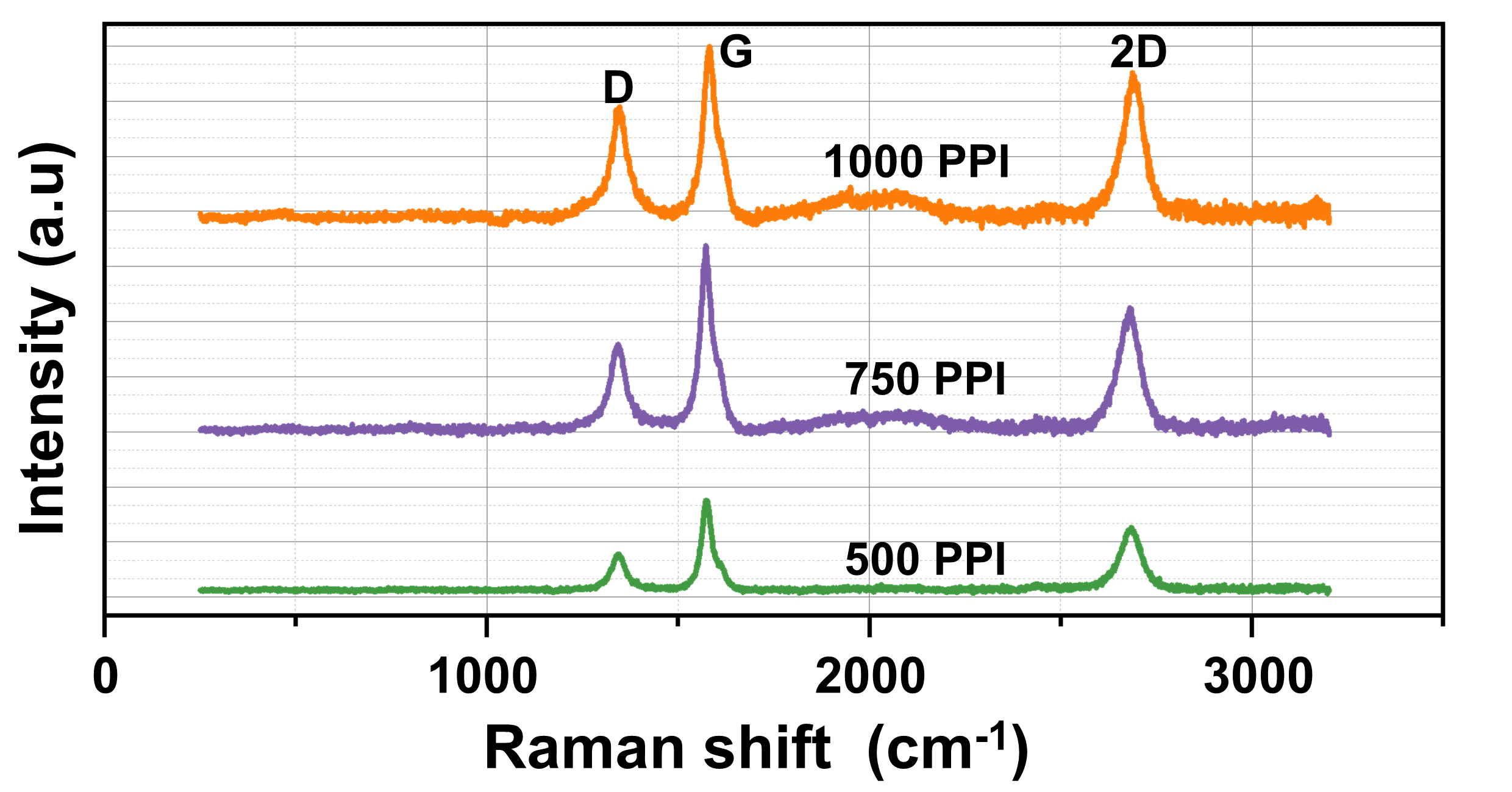


**Figure S7.** Raman spectra of the LIG prepared by varying image density.


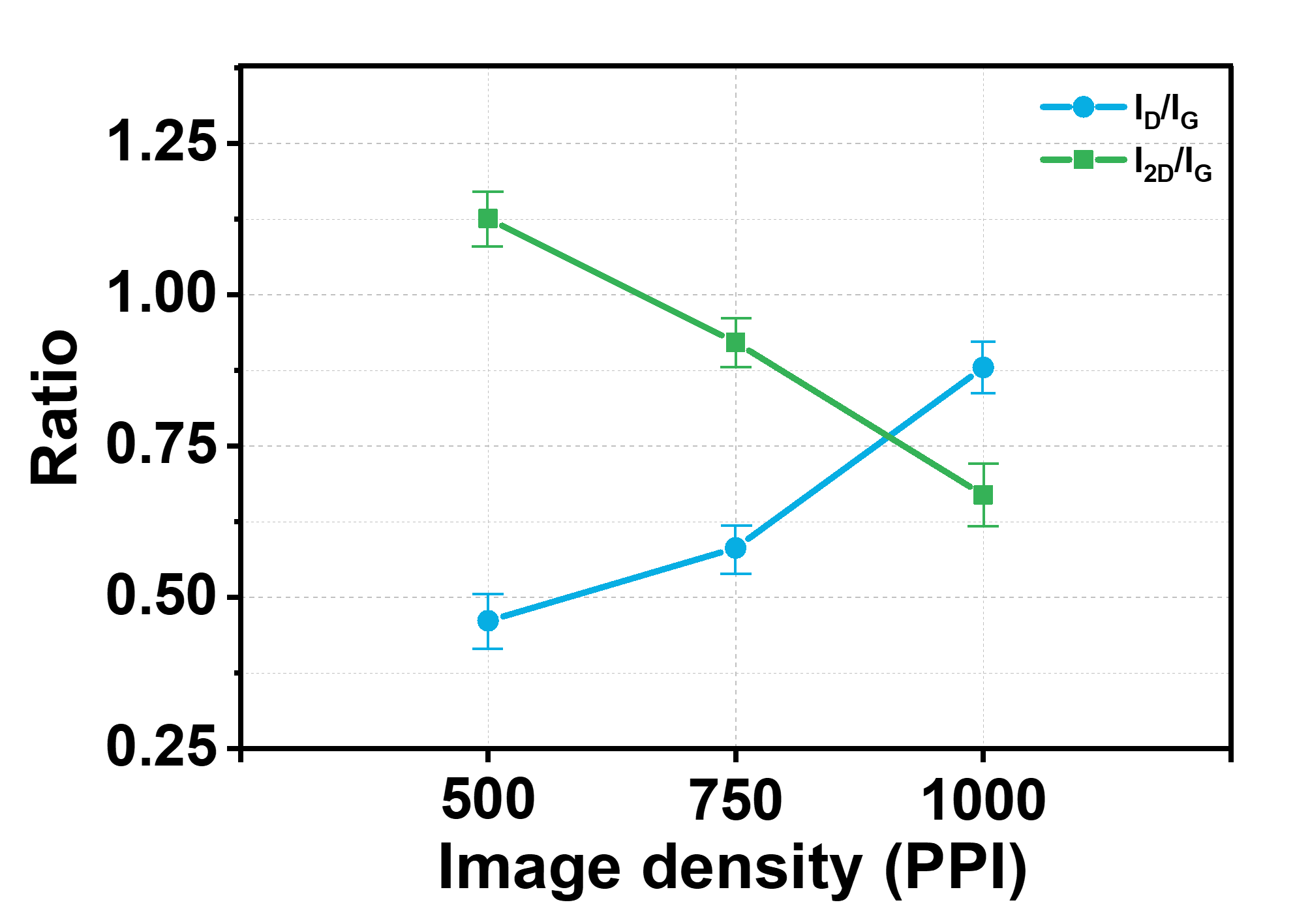


**Figure S8.** Analysis of the peak intensity ratios of the D and 2D to G obtained from the Raman spectra of the LIG prepared by varying image density.


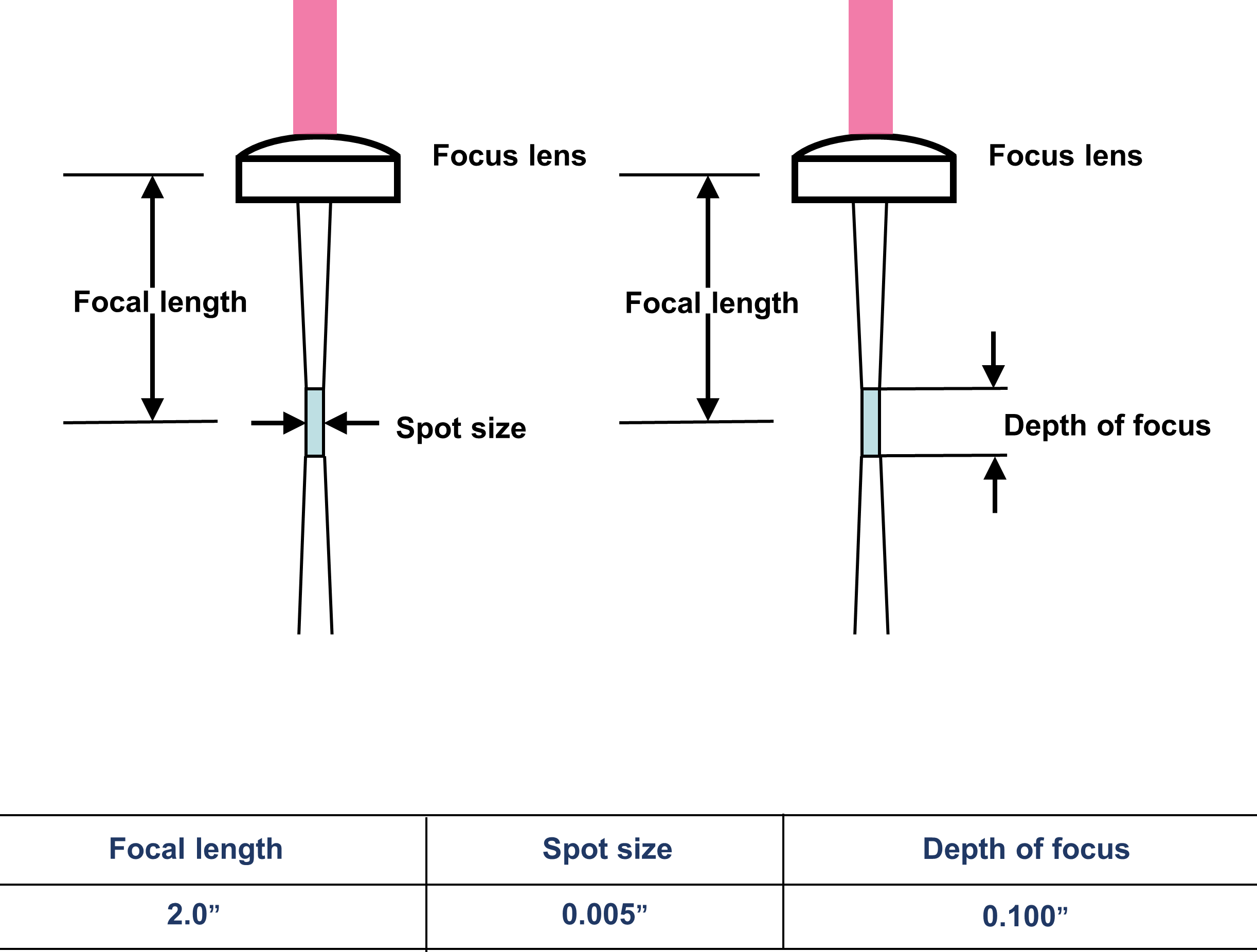


**Figure S9.** Diagram to show the spot size and depth of focus for the commercial CO_2_ laser system.


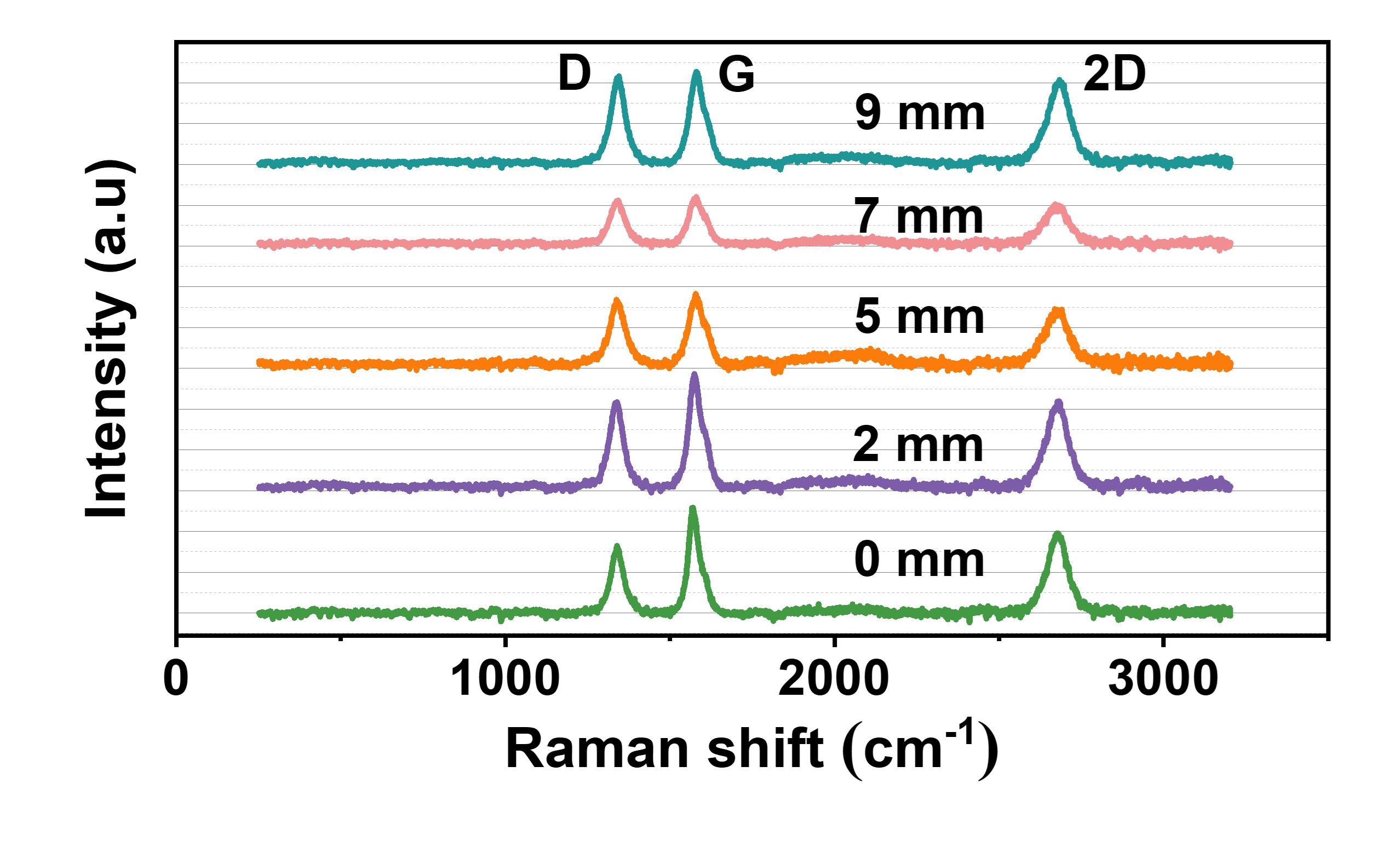


**Figure S10.** Raman spectra of the LIG prepared by varying defocus distances.


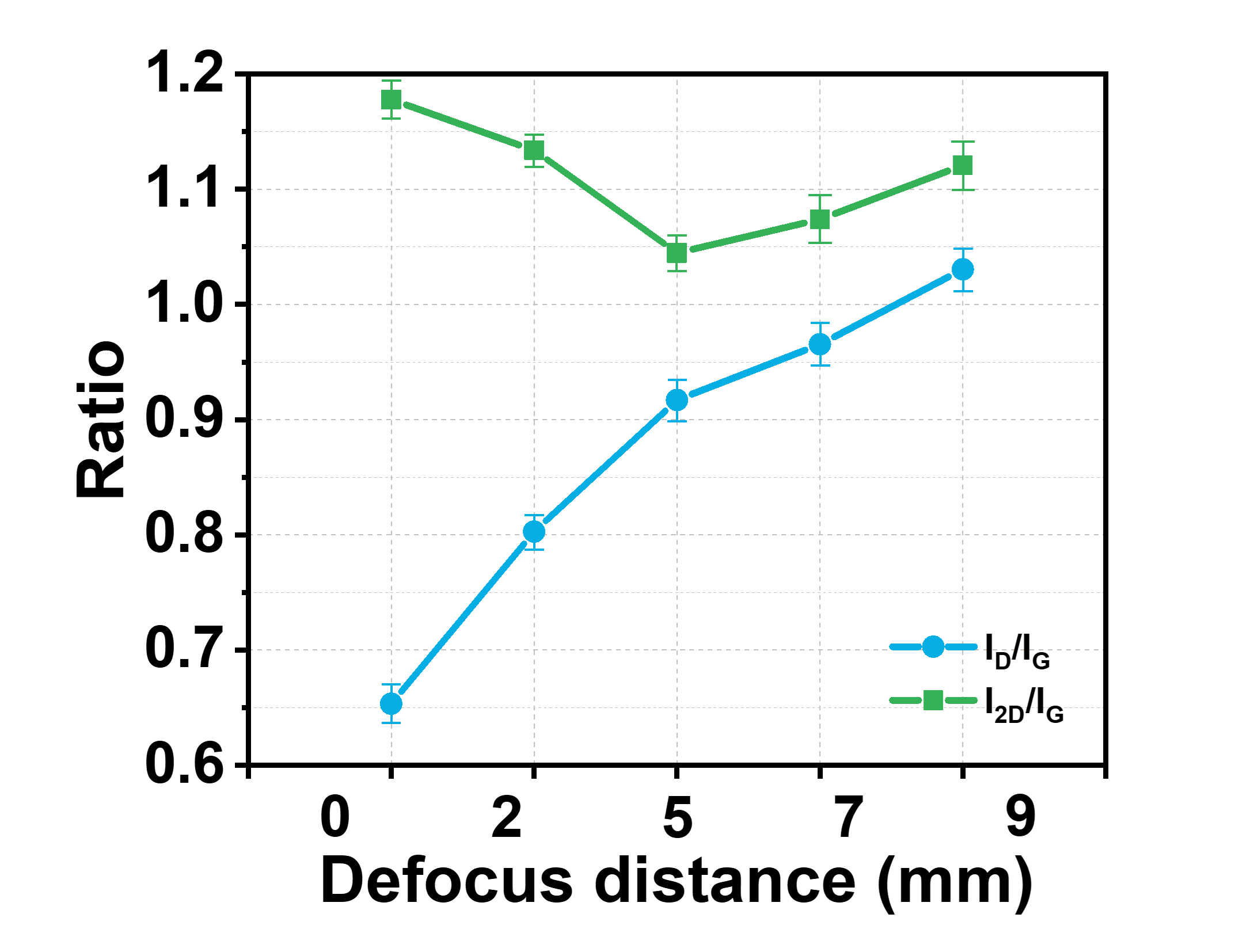


**Figure S11.** Analysis of the peak intensity ratios of the D and 2D to G obtained from the Raman spectra of the LIG prepared by varying defocus distances.


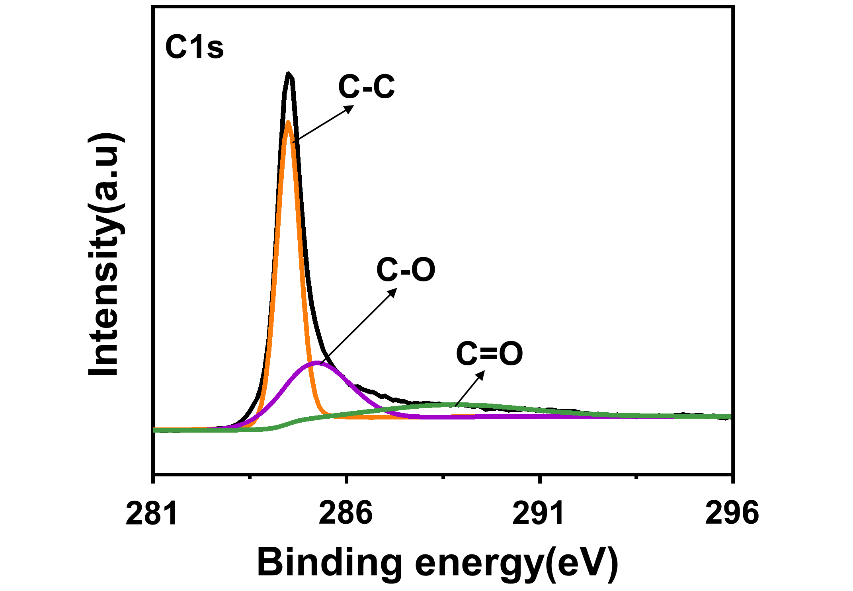


**Figure S12.** Deconvoluted C1s of the LIG prepared with the optimal laser processing parameters (i.e., laser power of 0.6 W, image density of 500 PPI, scanning speed of 2.54 mm/s).


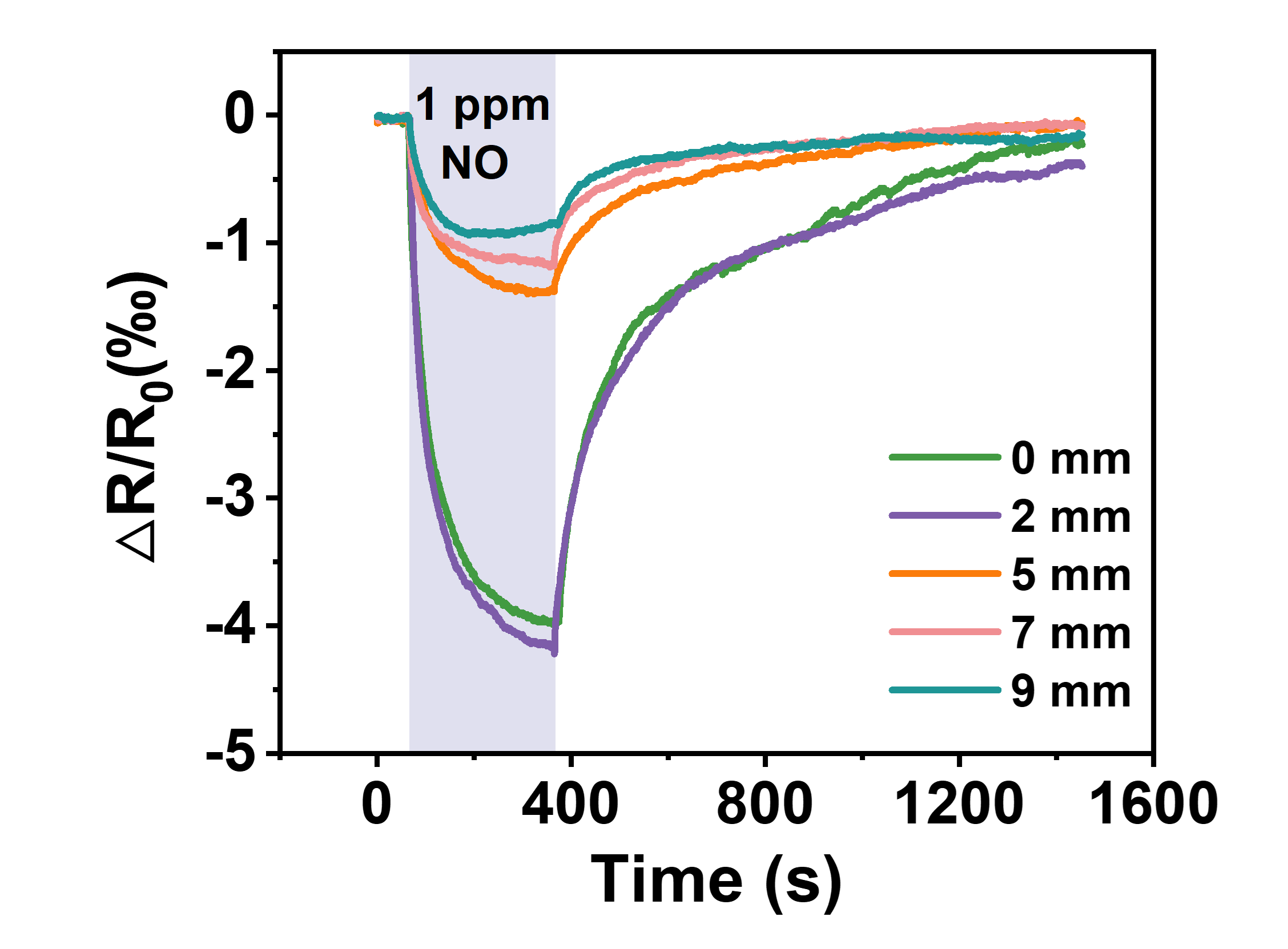


**Figure S13.** Gas sensing performance of the LIG-based gas sensors prepared with varying defocus distances.


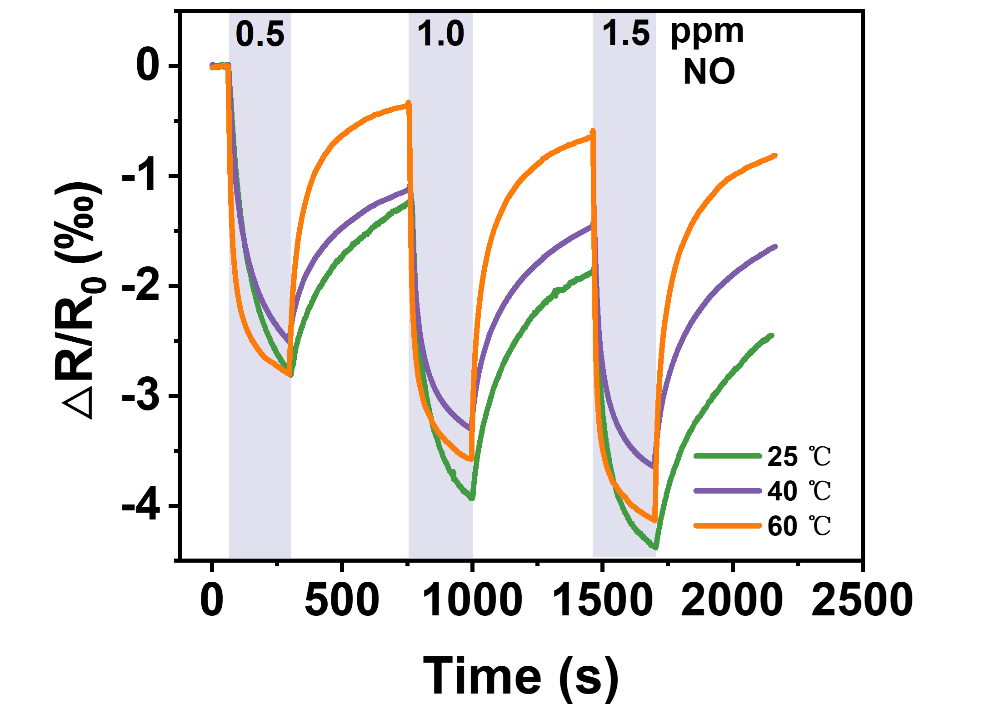


**Figure S14.** Dynamic response test of the LIG-based gas sensor without a semipermeable membrane at a testing temperature of 25 ℃, 40 ℃, and 60 ℃, which is created by an applied voltage of 0.05 V, 10 V, and 15 V.


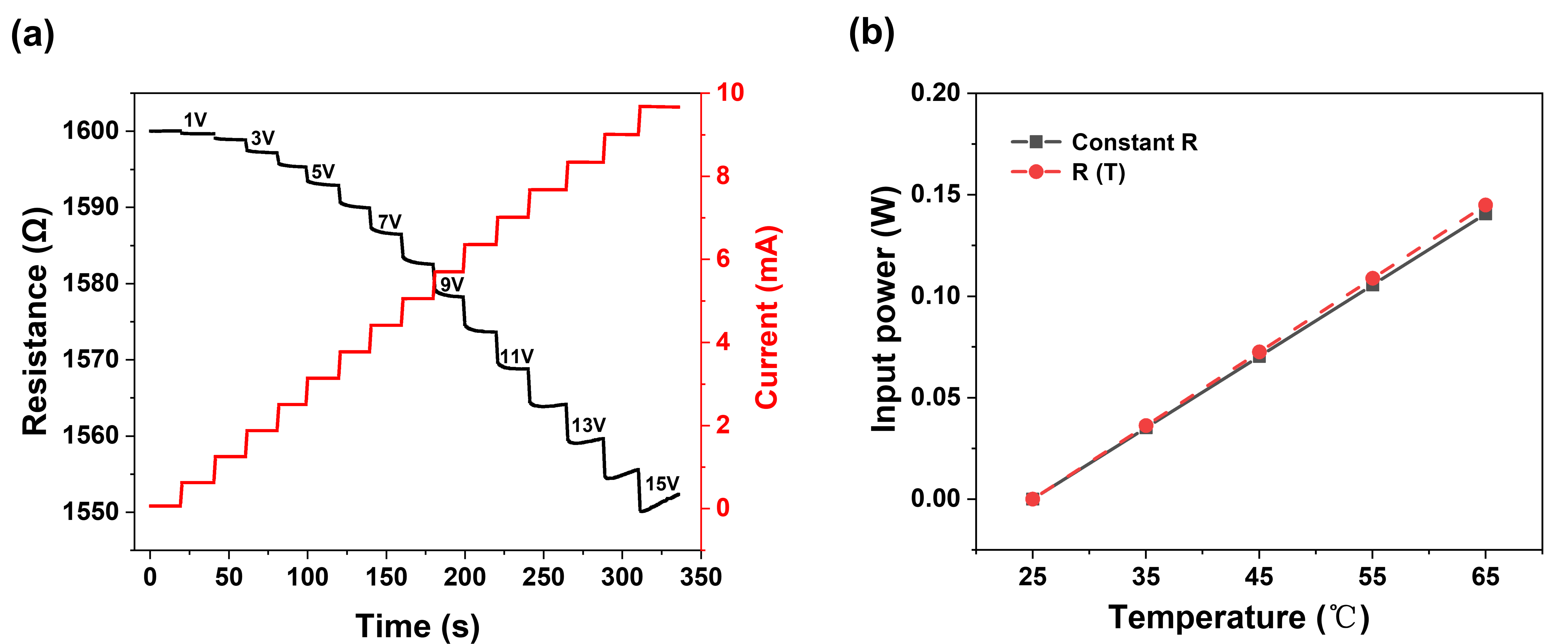


**Figure S15.** (a) Variation of the resistance and current in the LIG electrodes as the applied voltage is stepwise increased to 15 V. (b) Dependence of the temperature in the LIG electrode from self-heating on the input power, in which the solid black line assumes a constant resistance over time and the dashed red line uses the real-time resistance that decreases with the increasing temperature.


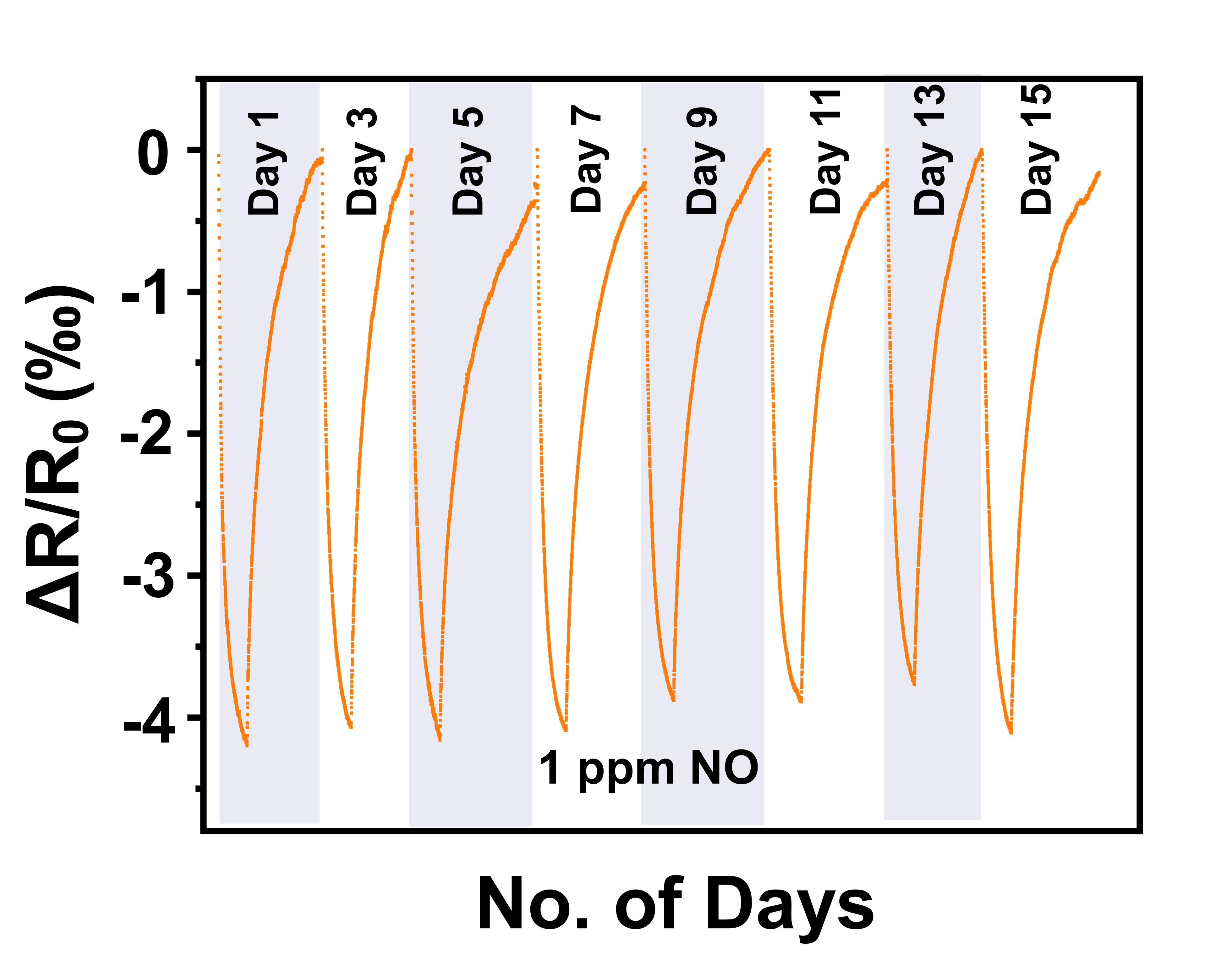


**Figure S16.** Long-term stability of the gas sensor to 1 ppm NO over 15 days.


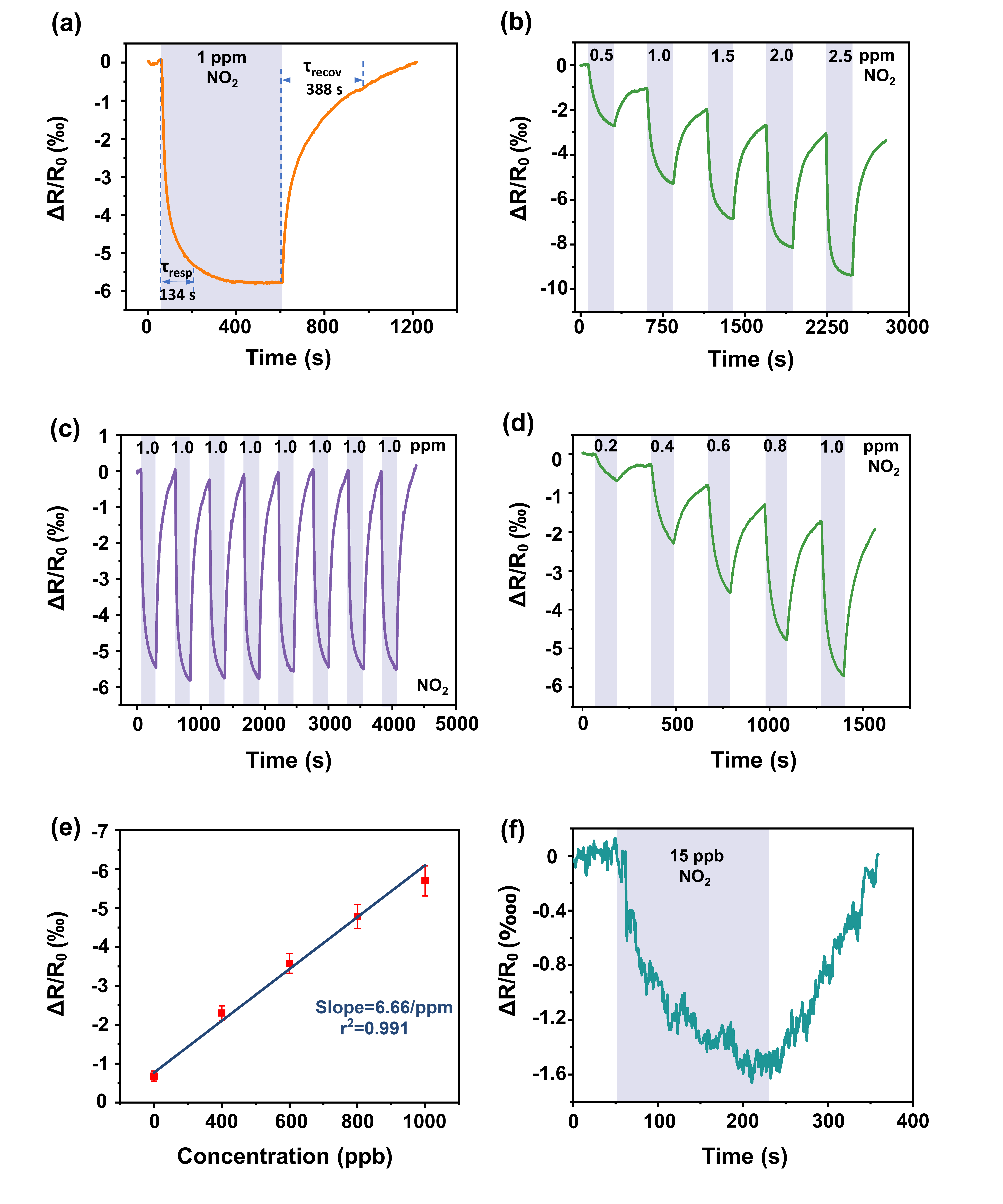


**Figure S17.** Gas sensing performance evaluation of the needle-like LIG-based gas sensor. (a) Typical response curve of the gas sensor to 1 ppm NO_2_. (b) Dynamic response curves of the gas sensor to NO_2_ with the concentration increasing from 0.5 to 2.5 ppm. (c) Repeatability test of the gas sensor to 1 ppm NO_2_ for eight consecutive cycles. (f) Dynamic response curves of the gas sensors to NO_2_ with the concentration increasing from 0.2 to 1.0 ppm. (e) Calibration curve with a linear fit obtained from the sensor response to NO_2_ from 200 to 1,000 ppb. (f) Experimental demonstration of the sensor response to 15 ppb NO_2_.


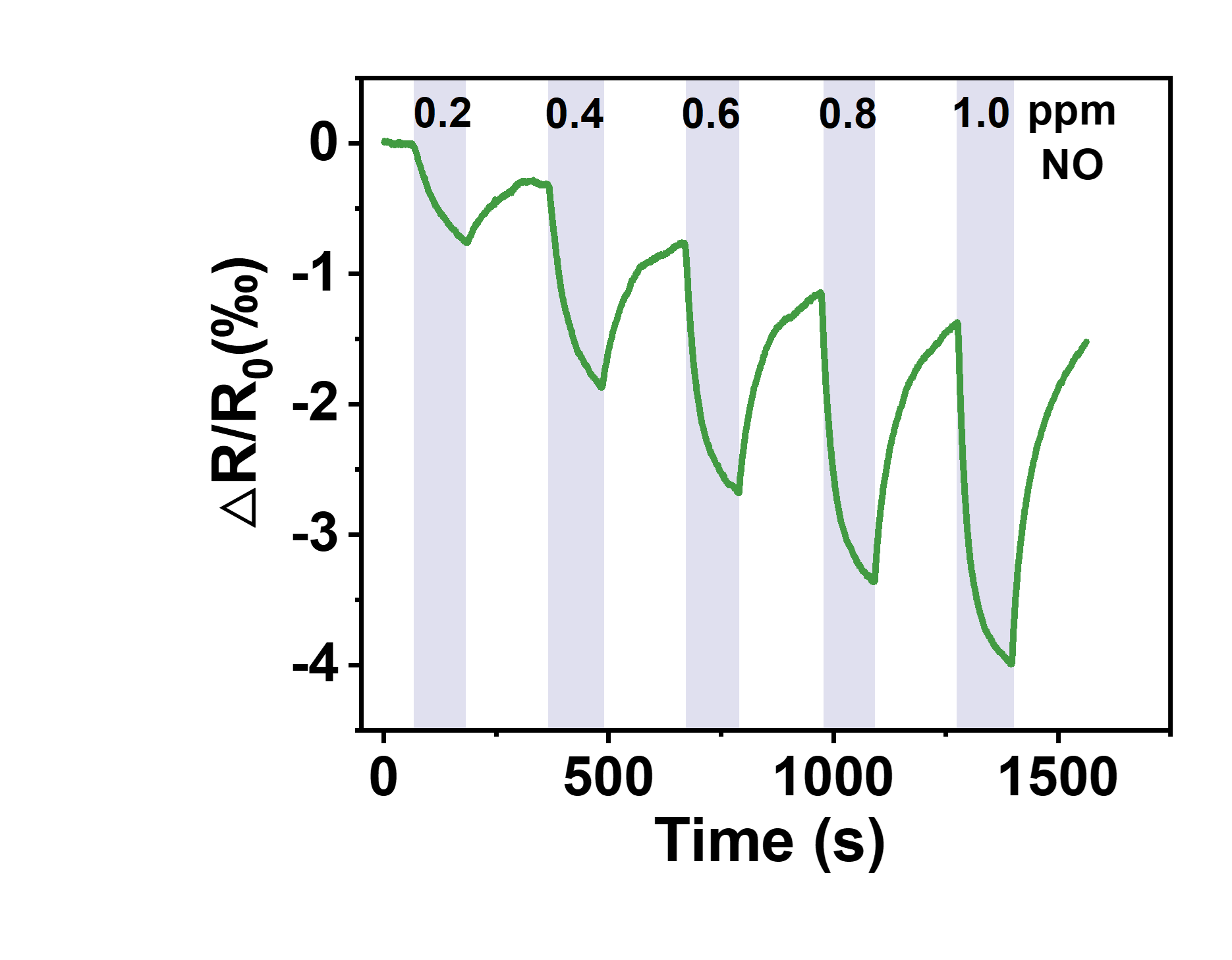


**Figure S18.** Dynamic response curves of the gas sensors to NO with the concentration increasing from 0.2 to 1.0 ppm.


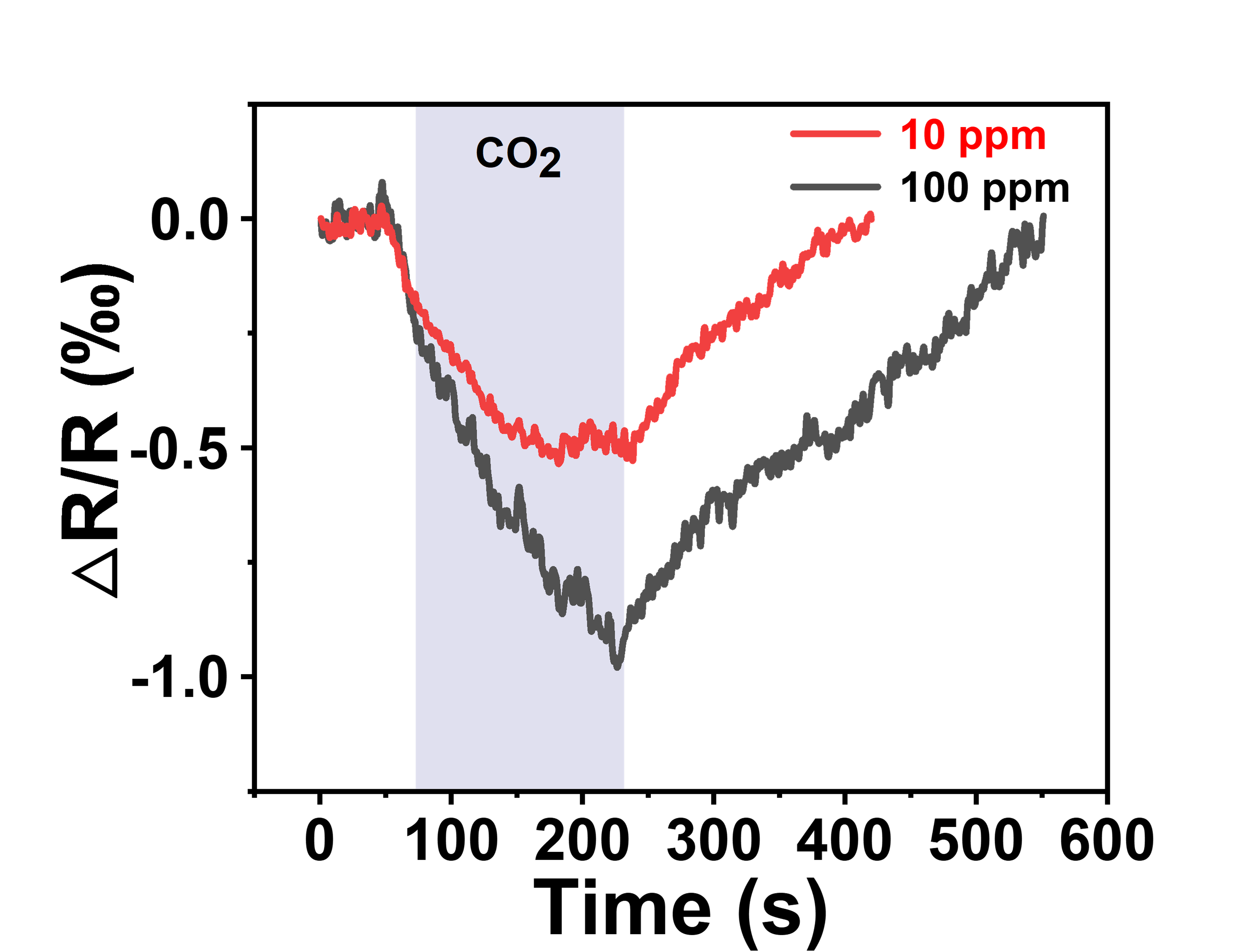


**Figure S19.** The response curve of the sensor to different concentrations of CO_2_.


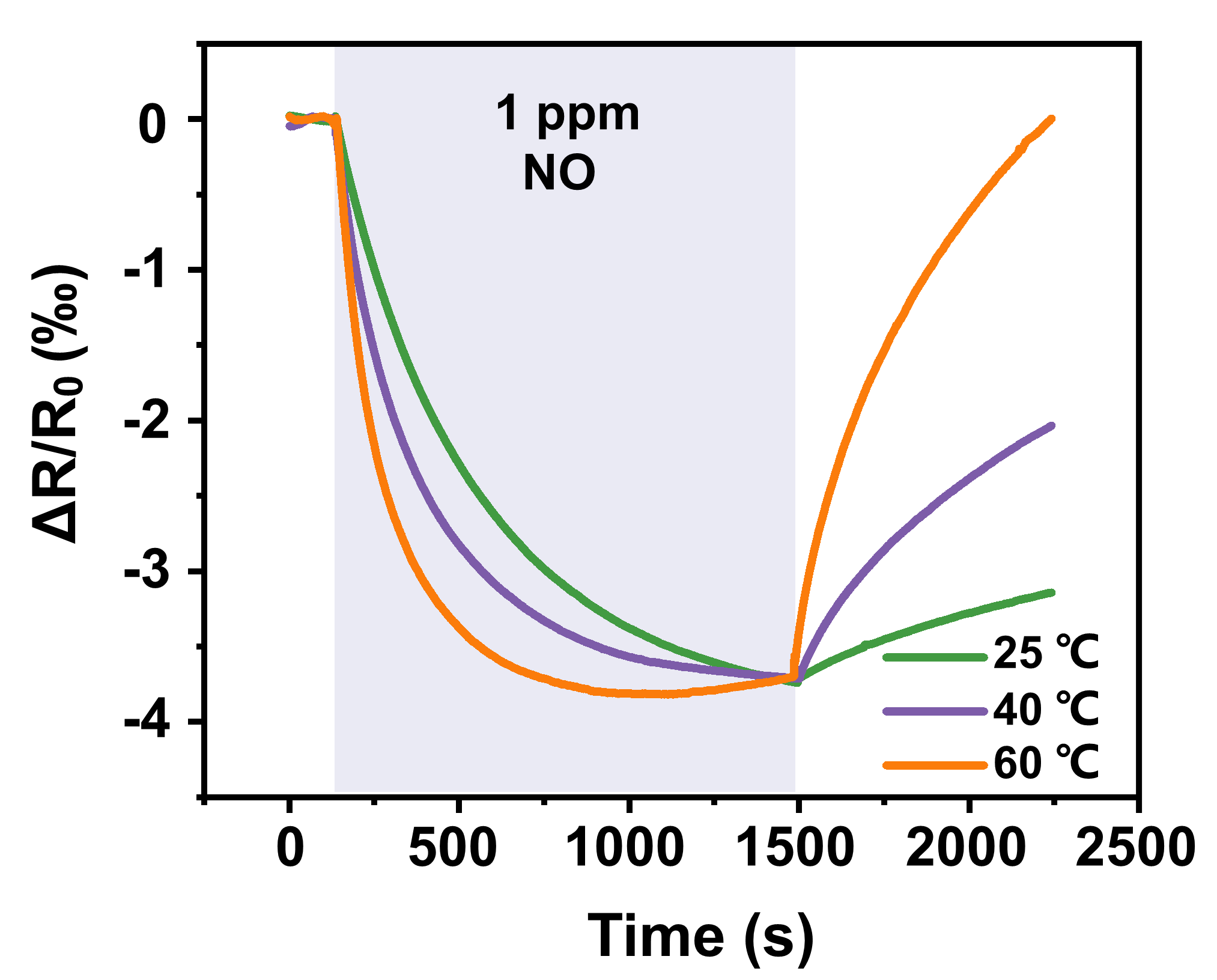


**Figure S20.** Response curves of the stretchable LIG-based gas sensor with a semipermeable membrane at different operating temperatures.


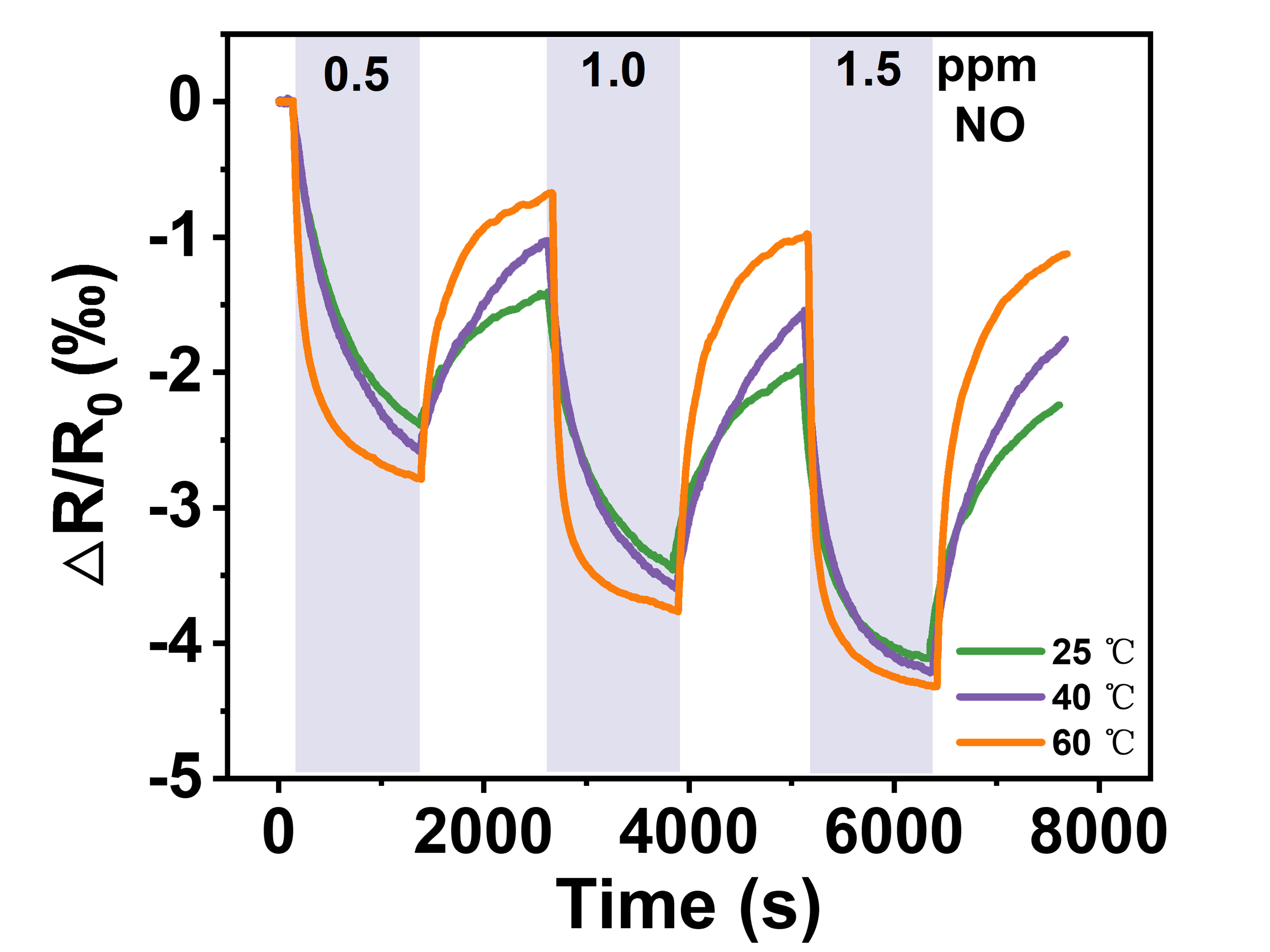


**Figure S21.** Dynamic response curves of the stretchable LIG-based gas sensor with a semipermeable membrane at different operating temperatures.


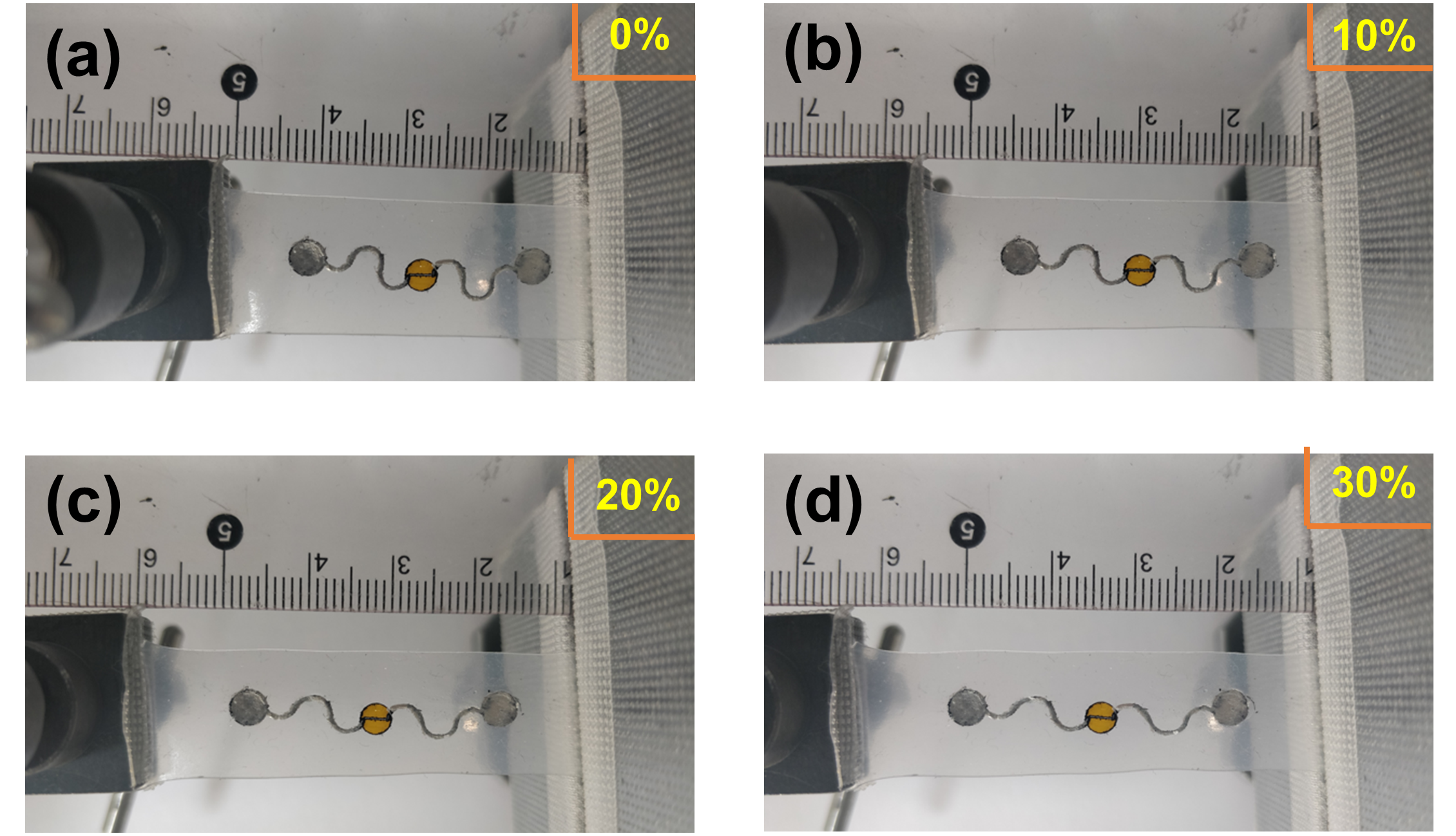


**Figure S22.** Optical images of the stretchable LIG-based gas sensor under varying uniaxial tensile strain levels: (a) 0%, (b) 10%, (c) 20%, and (d) 30%.


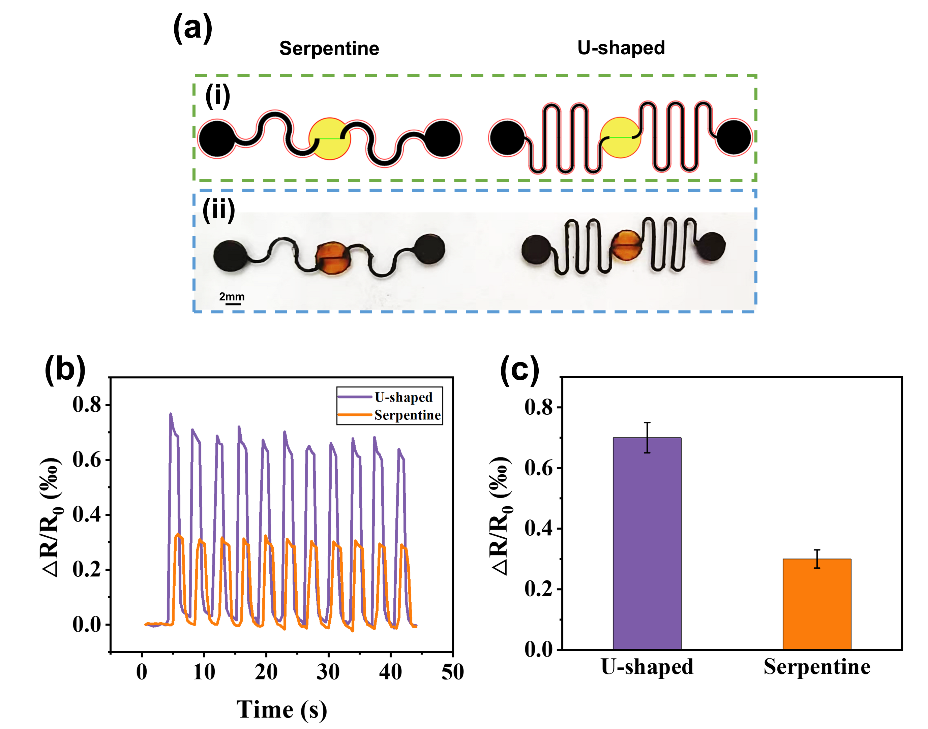


**Figure S23.** Stretchable LIG gas sensors with different electrode designs and their performance evaluations in the presence of cycling tensile strains. (a) Schematic illustration (top) and optical image (bottom) of the gas sensor with two different electrode designs. (b) Time-dependent resistance fluctuation and (c) average change of the resistance of the LIG gas sensors with U-shaped or serpentine electrode designs (uniaxial tensile strain of 30%).


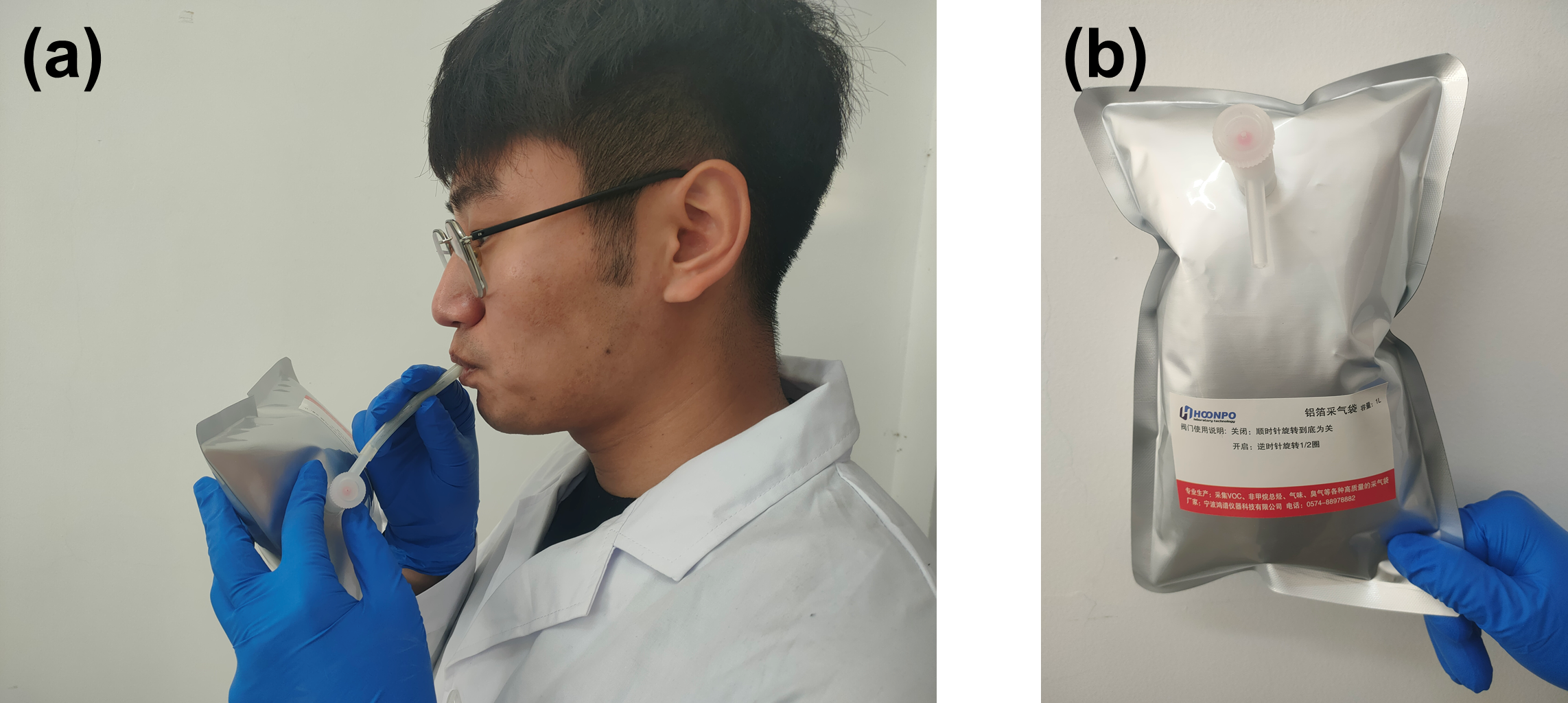


**Figure S24.** Optical images to show (a) the method for the collection of (b) the exhaled breath sample into an aluminum foil gas collecting bag.


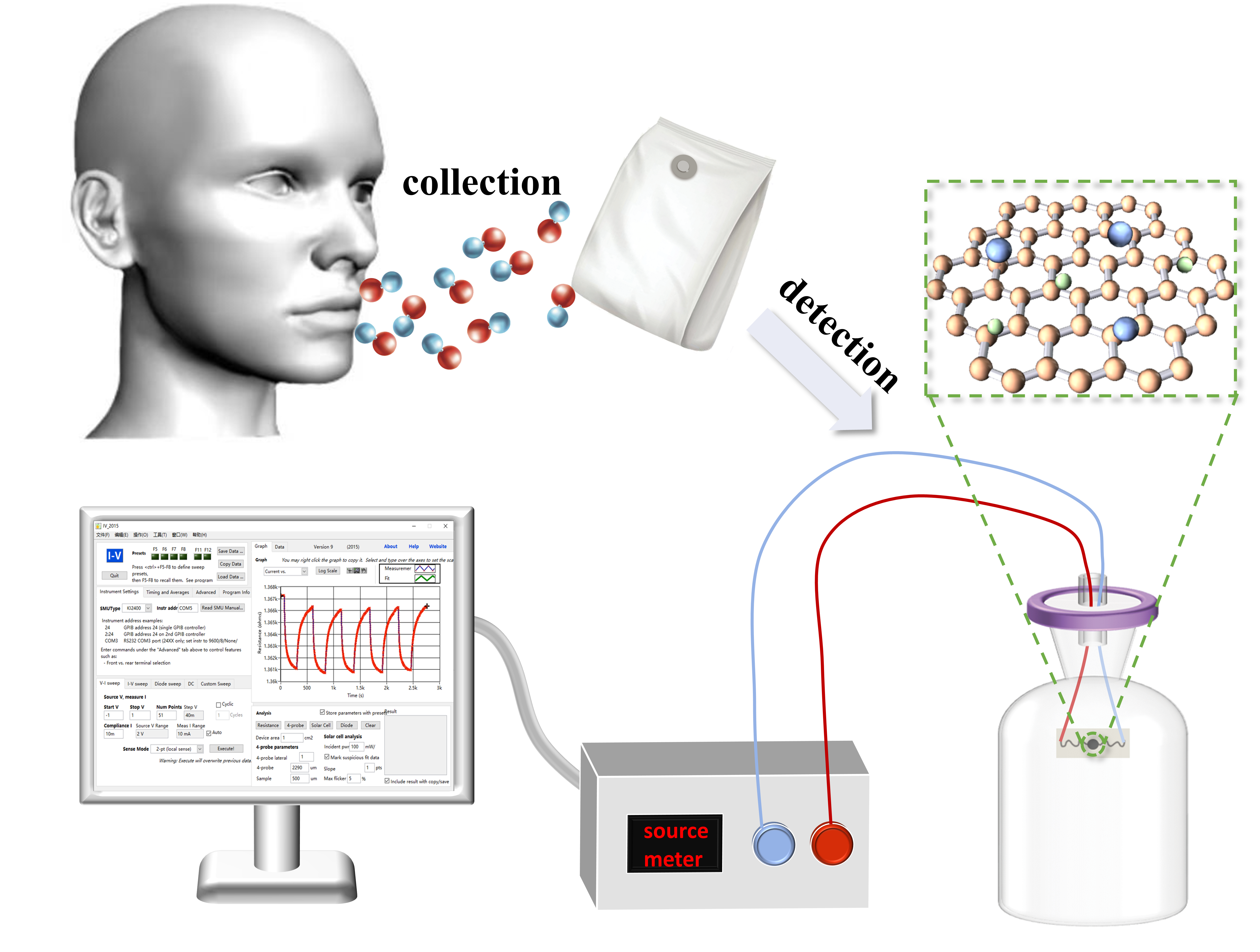


**Figure S25.** Schematic diagram to show the collection and detection of exhaled human breath samples.


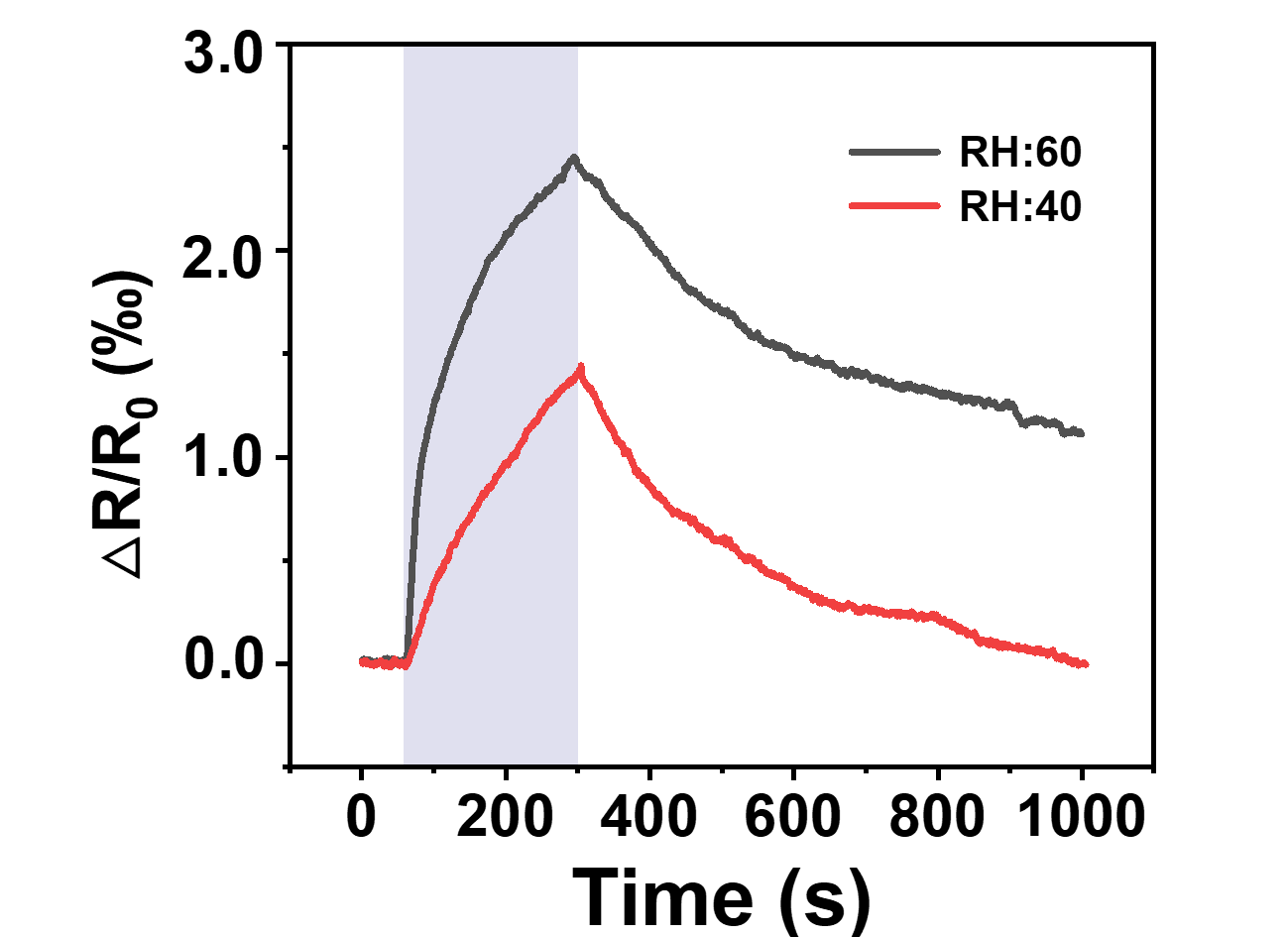


**Figure S26.** The response curve of the sensor in different humidity levels.


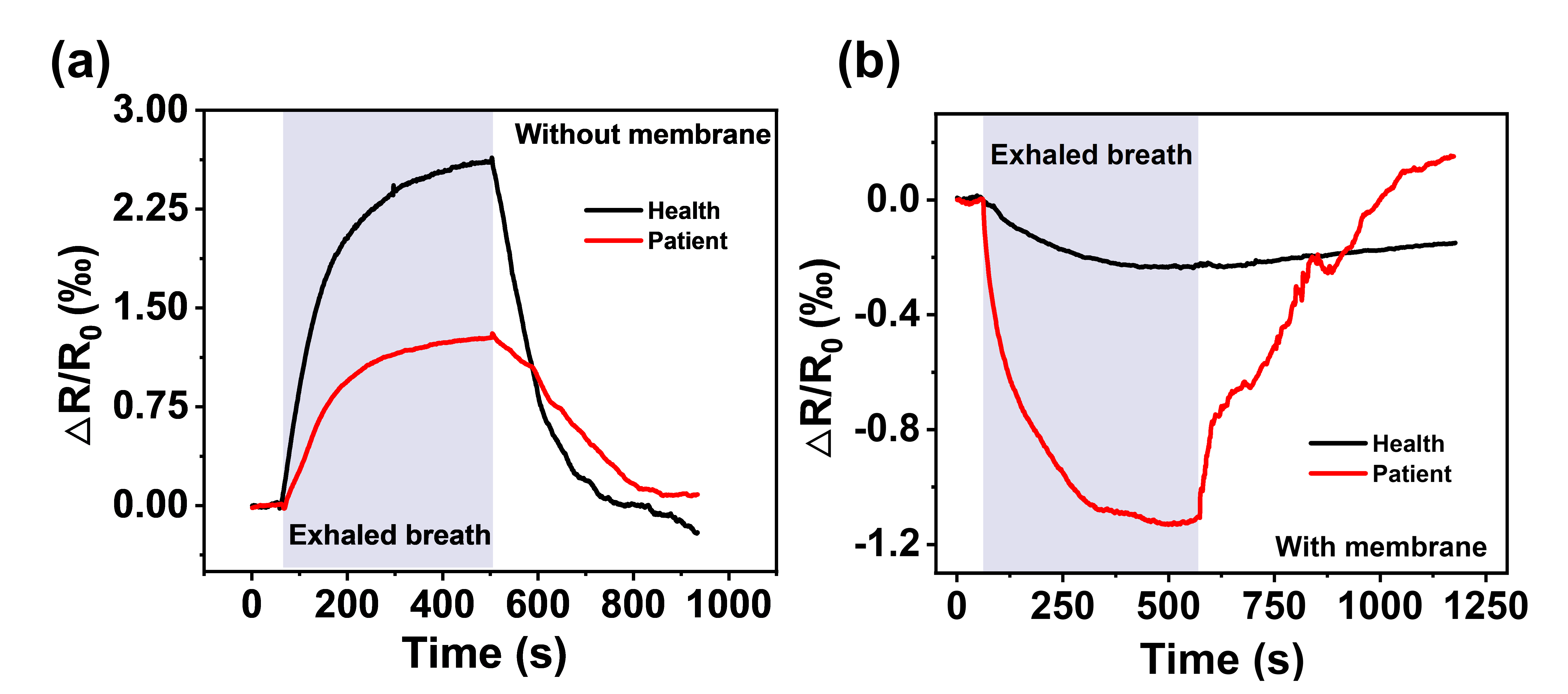


**Figure S27.** Typical response curves of the gas sensor (a) without and (b) with the semipermeable membrane to the exhaled human breath samples from healthy volunteers and patients with respiratory diseases.


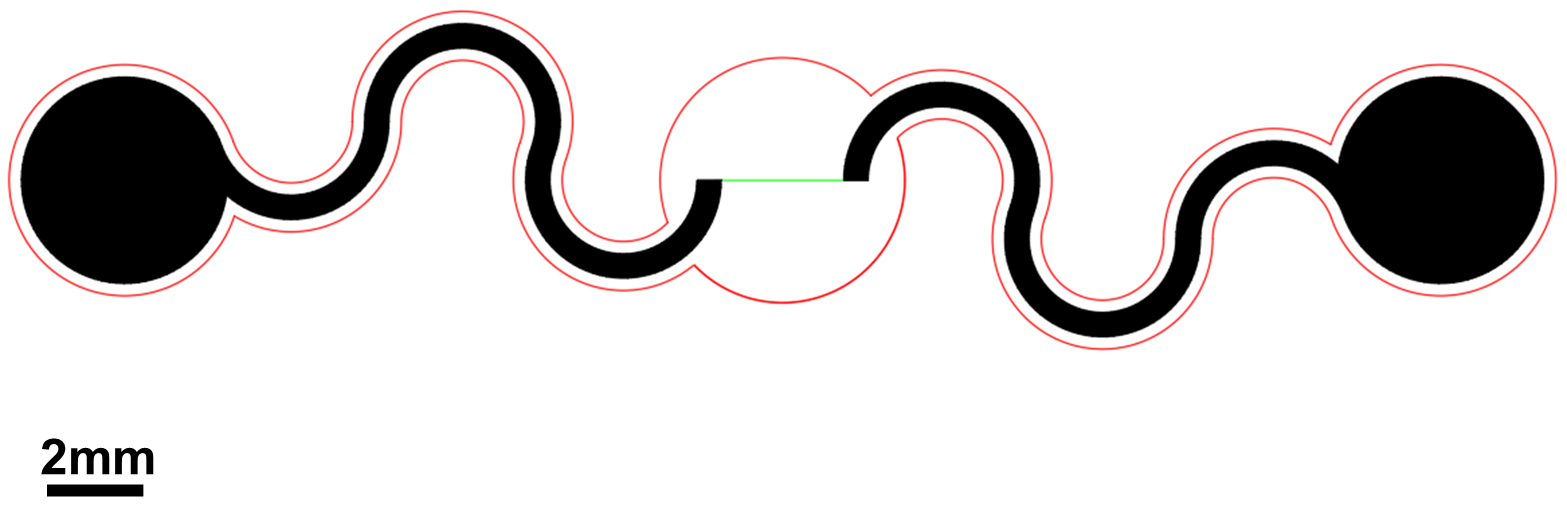


**Figure S28.** Designed LIG pattern (black: serpentine LIG electrode, green: Ag/LIG sensing region, and red: cutting line).

**Table S1.** Full width at half maxima (FWHM) and the peak intensity ratios of the D and 2D to G of the LIG prepared with varying laser powers.

|  | FWHM | | | Peak intensity ratio | |
| --- | --- | --- | --- | --- | --- |
| Peak  Power | D | G | 2D | D/G | 2D/G |
| 0.15 W | 46.7 | 55.1 | 81.5 | 1.30 | 0.86 |
| 0.60 W | 43.8 | 31.4 | 70.0 | 0.46 | 1.12 |
| 1.20 W | 55.3 | 63.3 | 76.4 | 0.96 | 0.84 |
| 1.80 W | 83.8 | 81.7 | 77.3 | 1.08 | 0.33 |

**Table S2.** FWHM and the peak intensity ratios of the D and 2D to G of the LIG prepared with varying image density.

|  | FWHM | | | Peak intensity ratio | |
| --- | --- | --- | --- | --- | --- |
| Peak  Image density | D | G | 2D | D/G | 2D/G |
| 500 PPI | 43.8 | 31.4 | 70.0 | 0.46 | 1.12 |
| 750 PPI | 46.0 | 35.5 | 61.1 | 0.58 | 0.92 |
| 1000 PPI | 51.8 | 40.6 | 38.9 | 0.88 | 0.67 |

**Table S3.** FWHM and the peak intensity ratios of the D and 2D to G of the LIG prepared with varying defocus distances.

|  | FWHM | | | Peak intensity ratio | |
| --- | --- | --- | --- | --- | --- |
| Peak  Defocus distance | D | G | 2D | D/G | 2D/G |
| 0 mm | 44.0 | 40.0 | 80.9 | 0.65 | 1.17 |
| 2 mm | 51.1 | 52.3 | 83.2 | 0.80 | 1.13 |
| 5 mm | 55.4 | 68.8 | 84.4 | 0.92 | 1.04 |
| 7 mm | 54.0 | 61.9 | 92.8 | 0.97 | 1.07 |
| 9 mm | 51.1 | 59.2 | 82.0 | 1.03 | 1.12 |

**Table S4.** Performance comparison between our LIG-based gas sensor and other NO_x_ gas sensors.

| Materials | Detection gas | Temperature (℃) | Response/recovery  Time (s) | LOD (ppb) | Flexible or stretchable | Moisture-resistant | Reference |
| --- | --- | --- | --- | --- | --- | --- | --- |
| MoS_2_ | NO | RT | 25/- (2 ppm) | 60 | No | No | Chen 2019^1^ |
| TiO_2_/rGO | NO | RT | 440/881 (2.75 ppm) | 690 | No | No | Kuchi 2021^2^ |
| MoS_2_/rGO | NO_2_ | 90 | — | 150 | Flexible | No | Jung 2018^3^ |
| Nanoribbons of α-MoO_3_ | NO_2_ | 125 | 1110/1800 (1 ppm) | 24 | No | No | Li 2021^4^ |
| MXene Ti_3_C_2_T_X_/ZnO | NO_2_ | RT | 34/103 (100 ppm) | — | Flexible | No | Yang 2021^5^ |
| 1D ZnO | NO_2_ | 150-170 | 9/220 (100 ppm) | 5000 | No | No | Godse 2021^6^ |
| Isonicotinamide/rGO | NO_2_ | RT | — | 1000 | No | No | Umar 2022^7^ |
| WO_3_ nanoplates | NO_2_ | 100 | — | — | No | Yes | Shendage 2017^8^ |
| MoS_2_ | NO_x_ | RT | 1.6/15.7 (100 ppm NO_x_) | 100 | No | No | Ikram 2018^9^ |
| Silicon nanomembrane | NO_x_ | RT | 30/60 (5 ppm NO_2_) | 20 | Stretchable | Yes | Ko 2020^10^ |
| Graphene/Dopamine | NO_2_ | RT | 85/- (100 ppm) | 1000 | Flexible | Yes | Lee 2020^11^ |
| chitosan/rGO | NO_2_ | RT | — | 1000 | Flexible | Yes | Park 2022^12^ |
| n‑SnSe2/p-SnO/n-SnSe | NO_2_ | RT | 34/272 (5 ppm) | 115 | Flexible | No | Rani 2022^13^ |
| LIG/MoS_2_ | NO_2_ | RT | 172/- (10 ppm) | — | Flexible | No | Yan 2020^14^ |
| LIG/rGO/MoS_2_ | NO_2_ | Self-heating | 360/720 (1 ppm) | 1.2 | Stretchable (tensile strain of  20%) | No | Yang 2020^15^ |
| LIG/MoS_2_ | NO_2_ | Self-heating | — | 0.3 | No | Yes | Peng 2022^16^ |
| LIG/Ag | NO_2_ | RT | 40/291 (1 ppm) | 6.1 | Flexible | No | Yang 2022^17^ |
| LIG | NO_x_ | RT | 113/296 (1 ppm NO)  134/388 (1 ppm NO_2_) | 8.3 (NO)  4.0 (NO_2_) | Stretchable (tensile strain of  30%) | Yes | **This work** |

**Table S5.** Statistics of the tested human volunteers.

| No. | Age | Gender | Type |
| --- | --- | --- | --- |
| 1 | 37 | Female | asthma |
| 2 | 65 | Male | asthma |
| 3 | 26 | Male | asthma |
| 4 | 44 | Male | asthma |
| 5 | 72 | Female | COPD |
| 6 | 38 | Female | COPD |
| 7 | 58 | Female | COPD |
| 8 | 61 | Male | COPD |
| 9 | 54 | Male | COPD |
| 10 | 68 | Male | COPD |
| 11 | 39 | Male | asthma |
| 12 | 35 | Female | asthma |
| 13 | 30 | Female | asthma |
| 14 | 67 | Male | asthma and COPD |
| 15 | 71 | Male | COPD |
| 16 | 62 | Male | COPD |
| 17 | 39 | Male | asthma |
| 18 | 17 | Male | asthma |
| 19 | 38 | Female | asthma |
| 20 | 47 | Female | asthma |
| 21 | 31 | Female | asthma |
| 22 | 55 | Male | COPD |
| 23 | 60 | Female | COPD |
| 24 | 24 | Male | health |
| 25 | 24 | Male | health |
| 26 | 24 | Male | health |
| 27 | 23 | Female | health |
| 28 | 25 | Female | health |
| 29 | 24 | Male | health |
| 30 | 23 | Male | health |
| 31 | 22 | Female | health |
| 32 | 23 | Male | health |
| 33 | 23 | Male | health |
| 34 | 24 | Male | health |
| 35 | 22 | Male | health |

**References**

1. Chen, Y.-Z. *et al.* An indoor light-activated 3D cone-shaped MoS_2_ bilayer-based NO gas sensor with ppb-level detection at room-temperature. *Nanoscale* **11**, 10410–10419 (2019).
2. Kuchi, C., Naresh, B. & Reddy, P. S. In situ TiO_2_-rGO nanocomposite for low concentration NO gas sensor. *Ecs J Solid State Sc* **10**, 037008 (2021).
3. Jung, M. W., Kang, S. M., Nam, K. H., An, K. S. & Ku, B. C. Highly transparent and flexible NO_2_ gas sensor film based on MoS_2_/rGO composites using soft lithographic patterning. *Appl Surf Sci* **456**, 7–12 (2018).
4. Li, W. *et al.* Ultrasensitive NO_2_ gas sensors based on layered α‐MoO_3_ nanoribbons. *Adv Mater Technol-Us*, 2100579 (2021).
5. Yang, Z. *et al.* Flexible resistive NO_2_ gas sensor of three-dimensional crumpled MXene Ti_3_C_2_T_X_/ZnO spheres for room temperature application. *Sensors and Actuators B: Chemical* **326**, 128828 (2021).
6. Godse, P. R. *et al.* Hydrothermally grown 1D ZnO nanostructures for rapid detection of NO_2_ gas. *SN Applied Sciences* **3**, 1–18 (2021).
7. Umar, A. *et al.* Supramolecularly assembled isonicotinamide/reduced graphene oxide nanocomposite for room-temperature NO_2_ gas sensor. *Environmental Technology & Innovation* **2**, 102066 (2021).
8. Shendage, S. S. *et al.* Sensitive and selective NO_2_ gas sensor based on WO_3_ nanoplates. *Sensor Actuat B-Chem* **240**, 426–433 (2017).
9. Ikram, M. *et al.* Multilayer flower like MoS_2_ conjugated with thin layer In(OH)_3_ for high-performance NO_x_ gas sensor at room temperature. *J Alloy Compd* **735**, 1439–1448 (2018).
10. Ko, G. J. *et al.* Biodegradable, flexible silicon nanomembrane-based NO_x_ gas sensor system with record-high performance for transient environmental monitors and medical implants. *Npg Asia Mater* **12**, 1–9 (2020).
11. Lee, S. W. *et al.* Highly conductive and flexible dopamine-graphene hybrid electronic textile yarn for sensitive and selective NO_2_ detection. *Acs Appl Mater Inter* **12**, 46629–46638 (2020).
12. Park, H. et al. Flexible and disposable paper-based gas sensor using reduced graphene oxide/chitosan composite. *J Mater Sci Technol* **101**, 165–172 (2022).
13. Rani, S. et al. Temperature-Dependent n–p–n Switching and Highly Selective Room-Temperature n-SnSe_2_/p-SnO/n-SnSe Heterojunction-Based NO_2_ Gas Sensor. *Acs Appl Mater Inter* **14**, 15381–15390 (2022).
14. Yan, W. H. *et al.* Size-tunable flowerlike MoS_2_ nanospheres combined with laser-induced graphene electrodes for NO_2_ sensing. *Acs Appl Nano Mater* **3**, 2545–2553 (2020).
15. Yang, L. *et al.* Novel gas sensing platform based on a stretchable laser-induced graphene pattern with self-heating capabilities. *J Mater Chem A* **8**, 6487–6500 (2020).
16. Peng, Z. *et al.* A Multi-functional NO_2_ gas monitor and self-alarm based on laser-induced graphene. *Chem Eng J* **428**, 131079 (2022).
17. Yang, L. et al. Intrinsically Breathable and Flexible NO_2_ Gas Sensors Produced by Laser Direct Writing of Self-Assembled Block Copolymers. *Acs Appl Mater Inter* **14**, 17818–17825 (2022).
